# Improving Fast‐Charging Performance of Lithium‐Ion Batteries through Electrode–Electrolyte Interfacial Engineering

**DOI:** 10.1002/advs.202411466

**Published:** 2024-11-22

**Authors:** Seungwon Kim, Sewon Park, Minjee Kim, Yoonhan Cho, Gumin Kang, Sunghyun Ko, Daebong Yoon, Seungbum Hong, Nam‐Soon Choi

**Affiliations:** ^1^ Department of Chemical and Biomolecular Engineering Korea Advanced Institute of Science and Technology (KAIST) 291 Daehak‐ro Yuseong‐gu Daejeon 34141 Republic of Korea; ^2^ Department of Materials Science and Engineering Korea Advanced Institute of Science and Technology (KAIST) 291 Daehak‐ro Yuseong‐gu Daejeon 34141 Republic of Korea; ^3^ Samwha Paint Ind. Co., Ltd 178 Byeolmang‐ro, Danwon‐gu Ansan Gyeonggi‐do 15619 Republic of Korea

**Keywords:** cathode‐electrolyte interface, electrolytes, lithium‐ion batteries, solid‐electrolyte interphase, solvation structures

## Abstract

The solid‐electrolyte interphase (SEI) is a key element in anode–electrolyte interactions and ultimately contributes to improving the lifespan and fast‐charging capability of lithium‐ion batteries. The conventional additive vinyl carbonate (VC) generates spatially dense and rigid poly VC species that may not ensure fast Li^+^ transport across the SEI on the anode. Here, a synthetic additive called isosorbide 2,5‐dimethanesulfonate (ISDMS) with a polar oxygen‐rich motif is reported that can competitively coordinate with Li^+^ ions and allow the entrance of PF_6_^–^ anions into the core solvation structure. The existence of ISDMS and PF_6_^−^ in the core solvation structure along with Li^+^ ions enables the movement of anions toward the anode during the first charge, leading to a significant contribution of ISDMS and LiPF_6_ to SEI formation. ISDMS leads to the creation of ionically conductive and electrochemically stable SEI that can elevate the fast‐charging performance and increase the lifespan of LiNi_0.8_Co_0.1_Mn_0.1_O_2_ (NCM811)/graphite full cells. Additionally, a sulfur‐rich cathode–electrolyte interface with a high stability under elevated‐temperature and high‐voltage conditions is constructed through the sacrificial oxidation of ISDMS, thus concomitantly improving the stability of the electrolyte and the NCM811 cathode in a full cell with a charge voltage cut‐off of 4.4 V.

## Graphical abstract

Synthetic isosorbide 2,5‐dimethanesulfonate is employed as an electrolyte additive that aids in the construction of interfacial layers to improve the fast‐charging kinetics and enable high‐temperature operation of high‐energy lithium‐ion batteries, surpassing the electrochemical performance of the conventional additive vinylene carbonate.

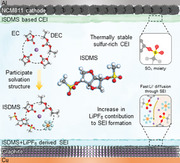

## 1 Introduction

Lithium‐ion batteries (LIBs) have fueled the advancement of portable devices over the past few decades and are currently the important drivers behind the growing electric vehicle (EV) industry. Ensuring a long lifespan and fast‐charging performance of high‐energy LIBs is vital for their widespread use as power sources for EVs.^[^ ^1^, ^2^, ^3^, ^4^ ^]^ Long‐lasting LIBs with a high energy density can be produced using high‐capacity cathodes, such as Ni‐rich cathodes and electrolytes with a wide electrochemical stability window.^[^ ^5^, ^6^, ^7^ ^]^ However, the implementation of fast‐charging characteristics for high‐energy LIBs remains challenging.^[^ ^8^, ^9^, ^10^ ^]^ A spatially dense and inhomogeneous solid‐electrolyte interphase (SEI) cannot ensure rapid and uniform Li^+^ ion transfer at high charging rates, which presents a critical impediment to the development of Li plating‐free graphite anodes that can suppress capacity decline and ensure the safety of LIBs.^[^ ^11^, ^12^ ^]^ The Li plating caused by limited ion permeation across the SEI is expected to be resolved by building an ionically conductive SEI through the sacrificial decomposition of additives.^[^ ^13^, ^14^, ^15^ ^]^ The conventional SEI modifier vinylene carbonate (VC) is employed to form a polymer‐like SEI with long‐term stability for extending the lifespan of some LIBs with graphite anodes. Fluoroethylene carbonate (FEC) is considered an appropriate additive to achieve excellent fast‐charging performance owing to the creation of a LiF‐rich SEI with polymeric/oligomeric species that can facilitate Li^+^ transfer across the SEI.^[^ ^16^, ^17^, ^18^, ^19^, ^20^ ^]^ Another additive, 1,3‐propane sultone (PS), can impart the cathode‐electrolyte interface (CEI) with good thermal stability by constructing a sulfur‐rich CEI.^[^ ^21^, ^22^ ^]^ However, PS produces a high‐resistance interfacial layer^[^ ^23^ ^]^ and VC makes the SEI dense and rigid, reducing the lithiation of the anode during fast charging and causing an undesired Li plating on the anode.^[^ ^24^ ^]^ Moreover, FEC undergoes corrosive HF generation that may damage the SEI and CEI and deteriorate the high‐temperature storage performance.^[^ ^25^ ^]^ Electrolyte additives that can simultaneously achieve long cyclability, high‐temperature storage performance, and fast‐charging capability have been developed.

Herein, we report a synthetic additive called isosorbide 2,5‐dimethanesulfonate (ISDMS), with a methyl sulfonate group based on the isosorbide skeleton, which improves the fast‐charging capability and high‐temperature stability of the interfacial layers of a LiNi_0.8_Co_0.1_Mn_0.1_O_2_ (NCM811)/graphite full cell. A high electron density of the oxygen atoms in the isosorbide skeleton of ISDMS enables complexing with Li^+^ ions while opening the possibility of PF_6_^–^ anions entering the core solvation structure and ingressing into the SEI. The high probability of the existence of PF_6_^–^ along with Li^+^ inside the SEI reduces the SEI resistance, facilitating Li^+^ ion transfer across the SEI, thereby realizing fast charging of LIBs under high current densities by facilitating subsequent charge transfer reactions. Moreover, the methanesulfonate group in ISDMS results in the generation of a sulfur‐rich CEI on the NCM811 cathode, enabling it to endure high‐temperature environments.

## 2 Results and Discussion

### 2.1 Synthetic Design of ISDMS as Electrolyte Additive

**Figure 1:**
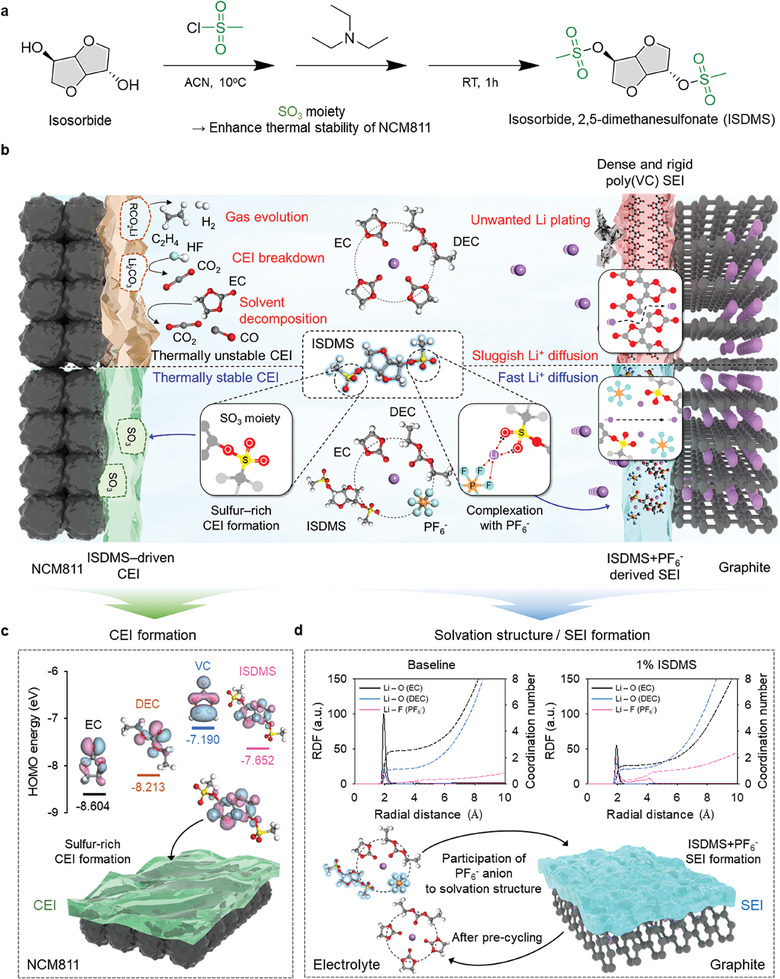
Schematic of the function of a synthetic additive ISDMS in an NCM811/graphite full cell. a) Synthesis of the ISDMS additive. b) Schematic of the features of ISDMS additive in an NCM811/graphite full cell. c) HOMO energy levels of EC, DEC, VC, and ISDMS and ISDMS‐driven CEI formation on the NCM811 cathode. d) Radial distribution function (RDF) and coordination number results of EC (C═O), DEC (C═O), and PF_6_^−^ (F) with respect to Li^+^ ion in the baseline and 1% ISDMS electrolytes, and ISDMS+PF_6_^−^‐derived SEI on the graphite anode.

The ISDMS additive was synthesized with a yield of 77% through mesylation and two nucleophilic attacks of the isosorbide skeleton (**Figure** **1**a). The SO_3_ motifs of ISDMS as thermally robust species act as a thermal enhancer of the interfacial layers on the electrodes, and oxygen atoms with nonbonding electrons are expected to be anti‐reductive SEI constituent that can endure the reductive environment at the anode during repeated charging processes.

The mesylation of methanesulfonyl chloride produces a sulfene structure. Subsequently, the two nucleophilic attacks of the isosorbide skeleton by alcohol produce an ISDMS additive (Figure S1, Supporting Information). From the ^1^H nuclear magnetic resonance (NMR) spectrum of the methanosulfonyl group, the strongest singlet peak can be identified at 3.1 ppm, which is most shielded owing to the presence of electron‐rich groups, such as oxygen and sulfur. Compared with the ^1^H NMR shift of the methanosulfonyl group, the ^1^H NMR peaks of hydrogen that are bonded to the carbon between the methanosulfonyl and isosorbide groups could be identified in the most de‐shielded area at 5.05 ppm. This is attributed to the reduced electron density from the oxygen atom in the isosorbide skeleton and the methanosulfonyl group (Figure S2, Supporting Information).

Figure 1b displays the beneficial role of the ISDMS additive in tuning the electrode–electrolyte interface structure for attaining high‐performance NCM811/graphite full cell with a LiPF_6_‐based electrolyte. The electrostatic potential mapping results of ISDMS suggest that a high electron density around the O atoms of the ISDMS molecules can help coordinate Li^+^ ion competitively with the EC solvent molecules (Figure S3, Supporting Information). In this regard, we believe that the resultant ISDMS‐incorporated complex has access. This makes it likely that ISDMS contributes to SEI formation along with PF_6_^−^ present in the solvation structure. The ISDMS‐driven, less‐resistive SEI can help facilitate the rapid movement of Li^+^ ions and thereby achieve the suppression of Li plating under fast‐charging conditions. Furthermore, ISDMS with a relatively high highest‐occupied molecular orbital (HOMO) energy level readily undergoes electrochemical oxidation, creating a thermally robust sulfur‐rich CEI on the NCM811 cathode, which leads to the alleviation of CEI breakdown at high temperatures (Figure 1c,d).

### 2.2 Electrochemical Behaviors of NCM811/Graphite Full Cells

Because high‐voltage operation is essential in ensuring a high‐energy LIB, high‐voltage stability of the electrolyte must be guaranteed. ISDMS exhibited relatively lower oxidation currents above 5.0 V versus Li/Li^+^ than VC, which is frequently used to form SEI on anodes in long‐lasting LIBs (Figure S4, Supporting Information). A synthetic additive ISDMS creates an appropriate interfacial layer to suppress electrolyte oxidation at high‐voltage cathodes. Further evidence that ISDMS suppresses electrolyte oxidation at high voltages can be inferred from the measurements of the leakage currents for NCM811/Li half cells by holding at voltages of 4.4, 4.5, and 4.6 V after pre‐cycling using an electrochemical floating test method (Figure S5, Supporting Information). The highest leakage current was detected in the baseline electrolyte, indicating acute oxidation of the electrolyte. Particularly, ISDMS discernibly reduced the leakage current caused by electrolyte oxidation at the cathode under high‐voltage conditions, suggesting the build‐up of an electrochemically robust CEI on the NCM811 cathode. The VC and ISDMS electrolytes exhibited improved discharge capacities of 194.4 and 195.7 mAh g^−1^ compared to the baseline electrolyte after pre‐cycling (Figure S6a,d,g, Supporting Information). The ISDMS electrolyte in the full cell showed an enhanced initial Coulombic efficiency of 89.2% relative to the baseline electrolyte (77.6%). This result implies that the SEI and CEI derived from ISDMS successfully mitigate the parasitic reactions of the electrolyte that undesirably consume Li ions and electrons at the cathode and anode during pre‐cycling (Figures S5 and S6b,e,h, Supporting Information). The SEI and CEI created by the VC and ISDMS electrolytes resulted in an increase in the impedance of the pre‐cycled NCM811/graphite full cell (Figure S6c,f,i, Supporting Information). Furthermore, 1wt. % ISDMS was found to be optimal content to ensure good cycle stability without a significant increase in electrolyte viscosity leading to the decrease in the ion conductivity of the electrolyte (Figures S7–S9, Supporting Information).

**Figure 2:**
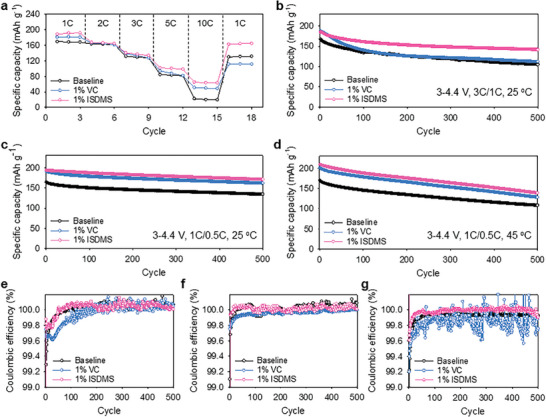
Electrochemical behaviors of NCM811/graphite full cells with different electrolytes: a) Charge rate performance of NCM811/graphite full cells at 25 °C. b) Fast‐charging cycle behaviors of NCM811/graphite full cells at 3 C charging and 1 C discharging rates. Cycle behaviors of NCM811/graphite full cells c) at 25 °C and d) 45 °C. Coulombic efficiencies of NCM811/graphite full cells during e) fast‐charging (3 C) cycling, f) cycling at 25 °C and g) cycling at 45 °C.

The cycle stability of the NCM811/graphite full cell was examined at various charging rates ranging from 1 C to 10 C to investigate the fast‐charging adaptability of the ISDMS electrolyte (**Figure** **2**a). In particular, the VC electrolyte had a capacity retention rate of 27%, whereas the ISDMS electrolyte showed an improved capacity retention rate of 34.8% at 10 C. In addition to the improved fast‐charging performance, the fast‐charging cycle stability of full cells with different electrolytes was evaluated at a charge rate of 3 C and a discharge rate of 1 C at 25 °C. The baseline and VC electrolytes showed capacity retention values of 62.8% and 59.7% while delivering discharge capacities of 105.0 and 111.7 mAh g^−1^, respectively (Figure 2b). However, the ISDMS electrolyte showed a much better capacity retention of 76.7% with a high discharge capacity of 141.8 mAh g^−1^. Because good fast‐charging cycle performance of full cells using the ISDMS electrolyte was achieved during extended cycling, it is believed that ISDMS is an effective interface modifier that protects the graphite anode and NCM811 cathode (Table S1, Supporting Information). Furthermore, improvements were also observed in the cycling performance at higher charging rates up to 5 C, exceeding that of 3 C (Figure S10, Supporting Information). During the fast‐charging (3 C) cycling, a disparity in the interfacial resistances between the ISDMS and VC electrolytes was observed as the cycle progressed. The VC electrolyte in the full cell showed a significantly increased resistance after 500 cycles (Figure S11, Supporting Information). Through the distribution of relaxation time analysis, it was confirmed that the increase in *R*_SEI+CEI_ was significantly lower in the ISDMS electrolyte than in the baseline and VC electrolytes during the fast‐charging (3 C) cycling of NCM811/graphite full cells (Figure S12, Supporting Information). Moreover, a disparity in the ohmic resistance of the full cell was demonstrated. Notably, the increase in the voltage of the full cell at a state of charge (SOC) level of 50% over 500 cycles was effectively mitigated in the ISDMS electrolyte (Figure S13, Supporting Information). This can be further confirmed by the reduced overpotential through the charge voltage profile for each cycle (Figure S14, Supporting Information). Thus, the ISDMS‐derived SEI and CEI are assumed to be electrochemically stable to ensure long‐term cycle stability under fast‐charging conditions. The ISDMS electrolyte showed noticeably improved capacity retention of 88.4% and 66.4% with discharge capacities of 170.7 and 138.9 mAh g^−1^ after 500 cycles at 25 and 45 °C, respectively (Figure 2c,d). The ISDMS electrolyte also demonstrated improved cycle performance during low‐temperature operations (Figure S15, Supporting Information). Moreover, the ISDMS electrolyte exhibited a stable and improved Coulombic efficiency, unlike the VC electrolyte, which showed a low and unstable Coulombic efficiency under a fast‐charge condition of 3 C and 45 °C (Figure 2e–g). Moreover, the enhancement of the fast‐charge cycle and high‐temperature (45 °C) cycle performance using the ISDMS electrolyte was observed in both the coin cells and pouch cells (Figure S16, Supporting Information). These outcomes represent that the ISDMS electrolyte enabled reversible electrochemical reactions at both the NCM811 cathode and graphite anode in the full cell while minimizing the parasitic reactions of the electrolytes, which may consume Li^+^ ions and electrons.

### 2.3 Fast‐Charging Characteristics of NCM811/Graphite Full Cells using ISDMS Electrolyte

**Figure 3:**
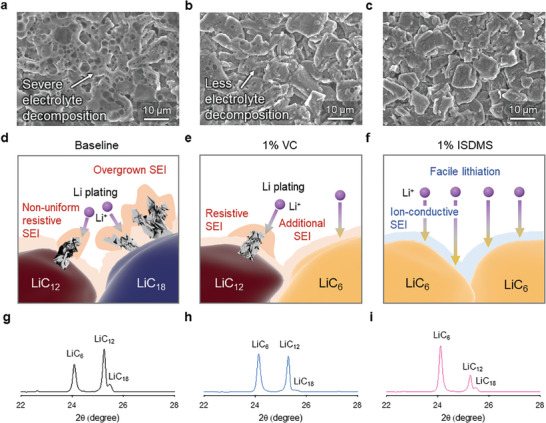
Morphological and structural changes of graphite anodes after the fast charging of the NCM811/graphite full cells. Top‐views of charged (lithiated) graphite at a charge rate of 5 C with a) baseline electrolyte, b) 1% VC electrolyte, and c) 1% ISDMS electrolyte. Schematic of the additional SEI evolution on Li plated on the top surface of graphite anodes with d) baseline electrolyte, e) 1% VC electrolyte, and f) 1% ISDMS electrolyte during fast charging. XRD patterns of charged (lithiated) graphite at a charge rate of 5 C with g) baseline electrolyte, h) 1% VC electrolyte, and i) 1% ISDMS electrolyte.

The adaptability of the ISDMS electrolyte on the fast‐charging characteristics of full cells was confirmed by investigating unwanted Li plating behavior on the graphite anode surface and crystal structures of lithium‐intercalated graphite after fast charging at 5 C. Since color changes in graphite indicate a change in the lithiation state of graphite, color change is a critical index for intuitively recognizing the phase transformation of graphite to Li_x_C_6_. Before the charging process, the pristine graphite anode was dark gray. Between SOC values of 30% and 50%, the graphite was dark blue. With further lithiation to an SOC range of 50%–90%, the graphite was dark red. Finally, over a SOC of 90%, the graphite turned gold.^[^ ^26^, ^27^, ^28^ ^]^ Graphite anodes after fast charging at 5 C in the baseline and VC electrolytes appeared to be dark gray, whereas the anode with the ISDMS electrolyte was dark red, indicating an increased lithiation of graphite (Figure S17, Supporting Information). This finding reveals that ISDMS electrolyte builds a more ion‐permeable SEI compared to that with the baseline and VC electrolytes. A noticeable feature is that the graphite particles with the baseline electrolyte could not be distinguished due to the locally and severely accumulated byproducts (**Figure** **3**a; Figure S18, Supporting Information). We believe that the highly resistive SEI hindered the intercalation of Li^+^ ions into graphite under fast‐charging conditions at 5 C. Therefore, unwanted Li plating in contact with the electrolyte was formed, resulting in further electrolyte decomposition around metallic Li (Figure 3a,b,d,e). In contrast, the ISDMS electrolyte showed a clean graphite surface with fewer byproducts than those of the VC electrolyte, which showed a dark gray graphite anode with severely localized accumulation of byproducts (Figure 3c,f; Figure S17, Supporting Information). Further evidence from X‐ray diffraction (XRD) patterns of the charged graphite anodes confirmed that the ISDMS electrolyte enabled the rapid lithiation of graphite anode at a charge rate of 5 C, leading to the appearance of a pronounced LiC_6_ peak and increased intensity ratios of the LiC_6_ and LiC_12_ peaks (Figure 3g–i). This improvement was also observed under extremely high‐charging conditions of over 5 C. The ISDMS electrolyte showed a clean anode surface with less Li plating even after charging at a rate of 10 C (Figure S19, Supporting Information). In contrast, the baseline and VC electrolytes failed to achieve complete lithiation of the graphite anode, inducing undesired Li plating that caused severe electrolyte decomposition. Additionally, the electrolyte decomposition by‐products covered the entire surface of the graphite anode, leading to the difficulty to distinguish the graphite particle boundaries. This finding suggests that the ISDMS‐derived SEI enables facile movement of lithium ions toward the graphite anode, leading to less entrapment of dead Li inside the SEI and Li plating on the top surface of the anode.

**Figure 4:**
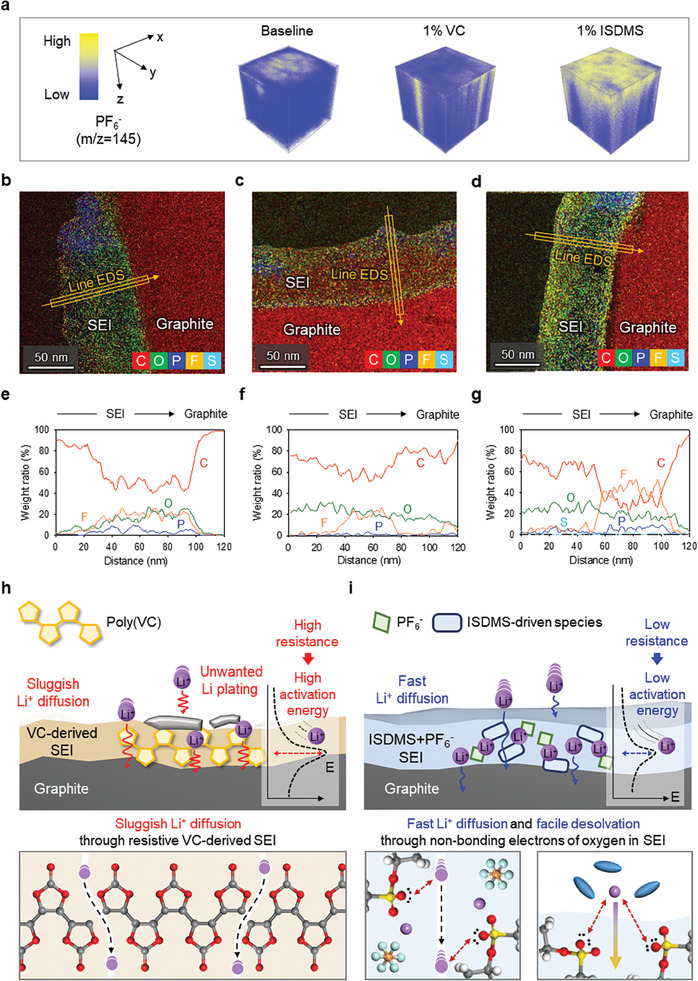
Surface chemistry of graphite anodes pre‐cycled with different electrolytes: a) TOF‐SIMS 3D cubic images of graphite anode after pre‐cycling. b–d) TEM images and EDS mapping results (red: carbon, green: oxygen, blue: phosphorus, yellow: fluorine, and sky blue: sulfur), and e‐g) line EDS‐weighted profile of graphite anode with b, e) baseline electrolyte, c,f) 1% VC electrolyte, and d, g) 1% ISDMS electrolyte after pre‐cycling. Schematic of Li^+^ diffusion crossing SEI on graphite anodes with h) 1% VC electrolyte, and i) 1% ISDMS electrolyte upon charging.

**Figure 5:**
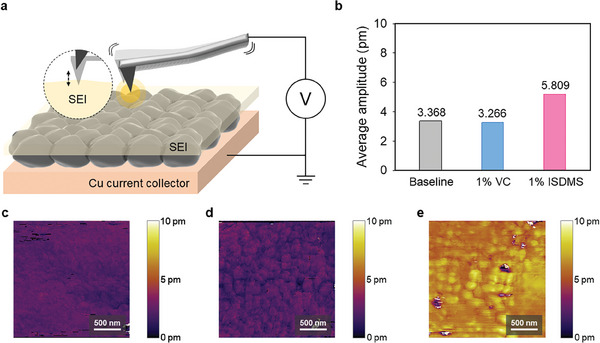
a) Schematic of the ESM measurement to determine the ion conductivity of the SEI. b) Calculated average amplitude of the SEI with different electrolytes after pre‐cycling at 25 °C. ESM amplitude images of SEI on the graphite anode pre‐cycled with c) baseline electrolyte, d) 1% VC electrolyte, and e) 1% ISDMS electrolyte.

To clarify the beneficial effect of ISDMS on the SEI for improving the fast‐charging performance, we examined the interface chemistry of the graphite anodes. Time‐of‐flight secondary ion mass spectroscopy (TOF‐SIMS) analyses of the graphite anodes after pre‐cycling revealed that the cell with the ISDMS electrolyte produced larger signals associated with PF_6_^−^ than the cells with the baseline and VC electrolytes (**Figure** **4**a). We inferred that the PF_6_^−^ anions present in the primary solvation structure contributed to SEI formation along with the ISDMS. Additional evidence of the participation of PF_6_^−^ in the creation of SEI was provided through X‐ray photoelectron spectroscopy (XPS). The appearance of the SO_3_^2−^ peak at 169.8 eV may be produced from the electrochemical reduction of ISDMS. Moreover, the relatively low proportion of LiF (684.7 eV, F 1s XPS) and the high fraction of P‐F species imply that ISDMS creates the electron‐rich polar SEI (Figure S20, Supporting Information). During pre‐cycling, the reductive decomposition of the C─O─C group of the isosorbide skeleton in ISDMS increased the intensity of the C─O peak at 533.5 eV in the O 1s XPS pattern (Figure S21a, Supporting Information). Furthermore, transmission electron microscopy (TEM) of graphite anodes after pre‐cycling revealed that the ISDMS formed uniform, thin SEI compared with the baseline and VC electrolytes. In addition, energy dispersive spectroscopy (EDS) data show that ISDMS‐derived SEI contains more F, P ratios, indicating an inorganic‐rich layer (Figure 4b–g; Figure S22, Supporting Information). Because no F atoms exist in the ISDMS, the F signal can originate from the decomposition of PF_6_^−^ at the graphite anode. The TOF‐SIMS depth profiling results revealed that the ISDMS electrolyte created SEI containing SO_3_^−^ species connected to carbon and Li. SEI created by the ISDMS electrolyte also contained P‐O species, which indicates the participation of the PF_6_^−^ anion in SEI formation (Figure S23, Supporting Information). The fast charging‐related issue lies in the momentary concentration of Li^+^ ions on the anode surface reaching zero, known as Sand's time, which induces Li nucleation and growth in specific regions.^[^ ^29^, ^30^, ^31^ ^]^ The reason why the ISDMS additive suppressed Li plating on the graphite anode under fast‐charging conditions can be explained using the SEI structure. The ISDMS contributed to the involvement of SO_3_ moiety, and PF_6_^−^ anions in the SEI construction, and the resulting polar species, such as P─F, P─O, and electron‐rich oxygen atom, not only facilitated the facile desolvation process but also provided lithium‐ion conduction pathways to alleviate the depletion of Li^+^ ions near the graphite anode (Figure S24a, Supporting Information).^[^ ^32^, ^33^, ^34^ ^]^ Moreover, unlike the dense and high‐resistance poly(VC)‐based SEI, the improvement of lithium‐ion transport kinetics across the SEI was enabled by decreasing the activation energy with the less compact and low‐resistance SEI structure, improving the fast‐charging capability of the NCM811/graphite full cell with less Li plating (Figure 4h,I; Figures S24b and S25, Supporting Information).^[^ ^35^, ^36^ ^]^ Electrochemical strain microscopy (ESM) analysis provides further evidence for ionically conductive ISDMS‐derived SEI through visualization of the distribution of Li^+^‐conducting channels.^[^ ^37^, ^38^ ^]^ Significantly higher amplitude signal was detected in ISDMS‐derived SEI compared to baseline and VC‐derived SEIs, indicating higher ion conductivity (**Figure** **5**). NMR analysis was conducted using 1 M of LiPF_6_ dissolved in diethyl carbonate (DEC) or ISDMS: DEC (1:9 wt. %) to elucidate the participation of ISDMS in the solvation structure. The results of the NMR analysis of 1 M LiPF_6_ in ISDMS:DEC (1:9 wt. %) showed that the ^7^Li NMR signals related to LiPF_6_ exhibited upfield shifts, whereas the ^19^F NMR peaks shifted to downfields (Figure S26, Supporting Information). ISDMS with Lewis‐base oxygen atoms induced de‐shielding of the F atoms in PF_6_^−^ and shielding of the Li^+^ ion, indicating that the incorporation of ISDMS in the electrolyte contributed to the participation of PF_6_^−^ in the core solvation structure (Figure S27, Supporting Information). Because the proximity of ISDMS to Li^+^ ions implies the entry of the ISDMS into the primary solvation structure, the ISDMS significantly contributes to the creation of anode SEI during pre‐cycling (Figure 1d).

Molecular dynamics (MD) simulations of the electrolytes with and without the ISDMS were performed to verify the entry of ISDMS and PF_6_^−^ into the core solvation structure (Figure S28, Supporting Information). Although the coordination number of the DEC molecule remained basically unchanged, the coordination number of the EC molecules with Li^+^ ion decreased, and PF_6_^−^ anions increased in number in the ISDMS‐containing electrolyte (Figure 1d). The decrease in the coordination number of EC for Li^+^ ions was mainly attributed to the entrance of the ISDMS into the core solvation structure. The presence of ISDMS molecules in a solvation circle at a distance of 5 Å from the Li^+^ ions could be confirmed by the MD simulation. When combined with the radial distribution function (RDF) results, three EC molecules and one DEC molecule coordinate with a Li^+^ ion in the baseline electrolyte, whereas a new solvation structure is constructed in which the ISDMS molecule and PF_6_^−^ anion are substituted for two EC molecules (Figure S29, Supporting Information).

The changes in the solvation structure were verified by performing ^7^Li and ^19^F NMR analyses of the ISDMS electrolyte. In the ISDMS electrolyte, the ^7^Li NMR peak shifted upfield and a down‐shift occurred, considering the peak of F in PF_6_^−^ in the ^19^F NMR. This indicates that ISDMS competes with the EC solvent in coordinating with the Li^+^ ions, and PF_6_^−^ ions enter the solvation shell, causing the EC solvent to be pushed out of the solvation structure (Figures S26 and S30, Supporting Information).^[^ ^39^ ^]^ The coordination ability of the ISDMS additive increased the shielding effect for Li^+^ ions, with the binding energy of Li^+^ ions being −222.78 kJ mol^−1^, compared to −189.88 kJ mol^−1^ for EC (Figure S31, Supporting Information). Moreover, the reduced peak intensity corresponding to coordinated EC and the increased relative fraction for coordinated PF_6_^−^ in the Raman spectra, and increased coordinated PF_6_^−^ proportion in FT‐IR spectra support the entrance of ISDMS and PF_6_^−^ into the core solvation structure, as indicated by the MD simulation results (Figures S32 and S33, Supporting Information).^[^ ^40^ ^]^ These results demonstrate that the involvement of ISDMS in the solvation structure of the electrolyte actively modifies the SEI structure, allowing for the contribution of the PF_6_^−^ anion to the formation of an SEI.

### 2.4 Thermally Stable CEI Generated by ISDMS

The HOMO and LUMO energy levels of the compound could predict the prior decomposition of electrolyte components. The synthetic additive ISDMS has a HOMO of −7.652 eV, which is higher than those of the solvents EC and DEC (Figure 1c; Figure S34, Supporting Information). This suggests that ISDMS could oxidatively decompose to form CEI on the NCM811 cathode during the first charging process. The XPS analysis showed that the ISDMS leads to the construction of a sulfonate‐based CEI. Owing to the sulfonyl group of the ISDMS, a signal from SO_3_^2−^, known as one of the thermally stable inorganic constituents, appeared at 169.8 eV in the S 2p XPS pattern (Figure S35a, Supporting Information). In addition, compared with the baseline electrolyte, the intensity of the LiF peak at 684.7 eV decreased to a similar level as that of VC (Figure S35b, Supporting Information). As seen in Figure S34, Supporting Information, PF_6_^−^ anion had a higher HOMO energy than solvents and additives. This indicates that it is prone to oxidative decomposition at the cathode. However, the ISDMS electrolyte enabled the entrance of PF_6_^−^‐ anions to participate in the core solvation structure, and thereby, PF_6_^−^ anions moved to the graphite anode during the initial charging process, contributing to the SEI construction. At this moment, the relative concentration of PF_6_^−^ on the cathode surface is lower than that on the anode, leading to less decomposition of PF_6_^−^ even at high HOMO energy levels. Consequently, this results in decreased formation of LiF on the CEI in the ISDMS electrolyte (Figure S36, Supporting Information). This result implies that the decomposition of LiPF_6_ and the generation of LiF on the cathode are insignificant in the ISDMS electrolyte. Furthermore, the ISDMS oxidatively decomposed at the cathode to create CEI, and therefore, the intensities of the C─O peak at 533.5 eV and the C═O peak at 532 eV in the O 1s XPS increased compared with the baseline electrolyte (Figure S21b, Supporting Information). Interestingly, differential scanning calorimetry heating curves showed a decrease in the exothermic heat of the fully delithiated NCM811 cathode with the ISDMS electrolyte compared to the cathode with the baseline electrolyte (Figure S37, Supporting Information). Moreover, in the ISDMS electrolyte, the open‐circuit voltage (OCV) drop of the NCM811/graphite full cells (coin‐type cells and pouch cells) charged up to 4.4 V was lower during storage at 60 °C than in the baseline and VC electrolytes (Figures S38 and S39, Supporting Information). Therefore, the capacity retention and recovery noticeably improved after storage for 30 d at 60 °C. Inductively coupled plasma optical emission spectroscopy showed reduced dissoluton of transition metal ions from the delithiated NCM811 cathodes with ISDMS‐driven CEI storing in the baseline electrolyte for 3 days at 60 °C. This result supports the high heat resistance of ISDMS‐driven CEI (Figure S40, Supporting Information) and demonstrates that the ISDMS electrolyte could help improve the high‐temperature storage characteristics of the NCM811/graphite full cells at 60 °C. Based on the thermogravimetric analyzer analysis of Li_2_CO_3_ and Li_2_SO_4_ compounds, we found that the S─O bond was more thermally stable than the C─O bond (Figure S41, Supporting Information). Moreover, because carbon is more electrophilic than sulfur, it is vulnerable to nucleophilic attack, causing bonds to break more easily.^[^ ^41^, ^42^ ^]^ This implies that thermally stable S─O species help to minimize CEI damage by HF attack, thereby suppressing the exposure of the cathode surface to the electrolyte and the unwanted reduction of Ni^4+^ to Ni^2+^ (Figure S42, Supporting Information).^[^ ^43^ ^]^ Thermally stable CEI was maintained stably at high temperatures, and exothermic reactions between the electrolyte and delithiated NCM811 cathode could be alleviated (Figure S37, Supporting Information). Additionally, the oxygen atoms with nonbonding electron pair in CEI stabilize Ni^4+^ at delithiated states through charge balance (Figures S43 and S44, Supporting Information).^[^ ^44^, ^45^ ^]^

### 2.5 Structural Stability of NCM811 Cathodes and Graphite Anodes with ISDMS Electrolyte

**Figure 6:**
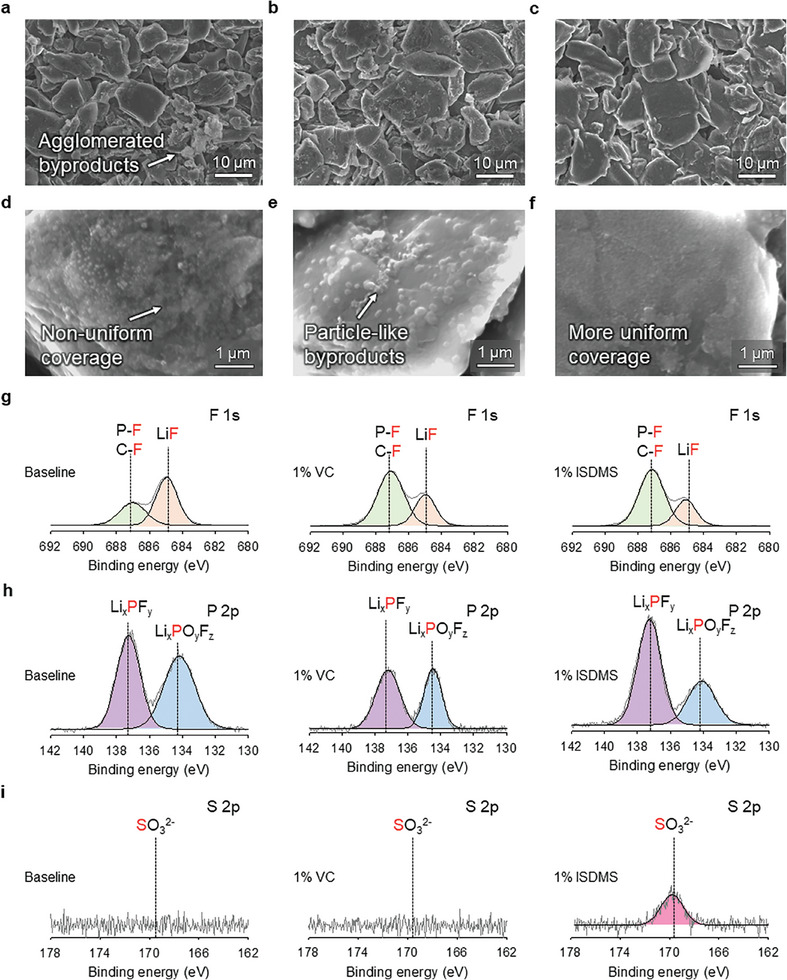
Surface analysis of graphite anodes from NCM811/graphite full cells after 500 cycles at 1 C/0.5 C and 25 °C. Surface morphologies of graphite anodes obtained from NCM811/graphite full cells cycled with a, d) baseline electrolyte, b,e) with 1% VC electrolyte, and c, f) with 1% ISDMS electrolyte. g) F 1s, h) P 2p, and i) S 2p XPS of graphite anodes in baseline, 1% VC electrolyte, and 1% ISDMS electrolyte after 500 cycles.

**Figure 7:**
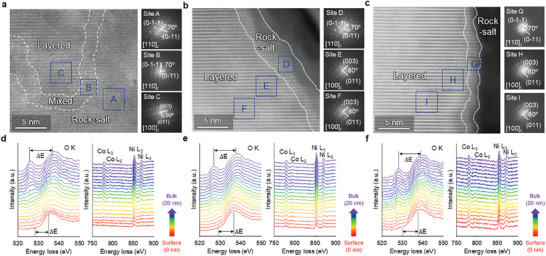
STEM images along with FFT patterns of NCM811 cathodes with a) baseline electrolyte, b) 1% VC electrolyte, and c) 1% ISDMS electrolyte after 500 cycles at 1 C/0.5 C and 25 °C. EELS spectra for O K‐, Co L‐, and Ni L‐edge from surface to the inner 500 cycled NCM811 particle with d) baseline electrolyte, e) 1% VC electrolyte, and f) 1% ISDMS electrolyte.

The SEM and XPS analyses helped verify the effect of the ISDMS on suppressing the interfacial deterioration of graphite anodes after 500 cycles at 1 C/0.5 C and 25 °C. The graphite anodes with the baseline and VC electrolytes had a bumpy surface with locally accumulated byproducts (**Figure** **6**a–c), whereas the ISDMS electrolyte made the graphite anode relatively smooth and clean. From the enlarged surface images depicted in Figure 6a–c, the difference in the surface morphology elucidated (Figure 6d–f). The graphite particles were uniformly covered with ISDMS+VC‐driven SEI, unlike that in the case of the baseline and VC electrolytes, leading to the creation of white, spherical‐like particles of various sizes on the surface of graphite particles. XPS analysis confirmed that this bumpy and rough anode surface after 500 cycles can be attributed to the generation of byproducts through continuous electrolyte decomposition. The P 2p and F 1s XPS patterns of graphite anode after cycling with the baseline electrolyte showed a high relative proportion of resistive LiF at 684.7 eV and Li_x_PO_y_F_z_ at 134.5 eV owing to LiPF_6_ decomposition, which reflects a similar trend observed in the data after pre‐cycling (Figure 6g,h; Figure S20, Supporting Information). These results indicate that the PF_6_^−^‐derived SEI by the ISDMS during the pre‐cycling process is well‐maintained after cycling, with no further LiPF_6_ decomposition (Figure S45, Supporting Information). However, nonuniformly distributed LiF grains generated via the decomposition of LiPF_6_ cannot provide effective channels for facile Li^+^ ion transport through LiF grains and may act as a resistive SEI component under high‐charging conditions (Figure S46, Supporting Information).^[^ ^46^, ^47^ ^]^ This explanation is rational in that the large proportion of resistive LiF in SEI created by the baseline electrolyte resulted in severe capacity fading at a high charge rate of 3 C (Figure 2b). In addition, the continuous decomposition of carbonate solvents led to a pronounced C═O signal at 532 eV as the SEI constituents on the graphite anode with the baseline electrolyte compared to the VC and ISDMS electrolytes (Figure S47a, Supporting Information). We believe that the use of sacrificial additives to form SEI on the graphite anode is an effective way to mitigate solvent decomposition during cycling. Notably, the S 2p XPS result shows that the SO_3_^−^‐containing species created by ISDMS decomposition remain present on the graphite anode, even after 500 cycles. This suggests that the long‐term stability of ISDMS‐promoted SEI ensures the minimization of parasitic reactions of the electrolytes at the anode (Figure 6i). Notably, the surface of the NCM811 cathode with the ISDMS electrolyte was clean without anchored insoluble byproducts compared with the cycled cathodes with the baseline and VC electrolytes (Figure S48, Supporting Information). The P 2p and F 1s XPS patterns show that relatively reduced proportions of LiF at 684.7 eV and Li_x_PO_y_F_z_ at 134.5 eV were observed for the NCM811 cathode cycled with the VC and ISDMS electrolytes (Figure S49a,b, Supporting Information). In addition, the C═O signal attributable to solvent decomposition at the cathode relatively decreased in intensity when the VC and ISDMS electrolytes were employed (Figure S47b, Supporting Information). Similar to the SEI structure formed on the graphite anode with the ISDMS electrolyte, the SO_3_‐related signal was detected as the CEI constituent on the NCM811 cathode (Figure S49c, Supporting Information). A noticeable feature in the surface morphologies of the NCM811 cathodes was that severe electrolyte decomposition occurred in the baseline and VC electrolytes. Consequently, a thick byproduct layer nonuniformly covered the primary cathode particles, making the primary cathode particles indistinguishable. On the contrary, in the ISDMS electrolyte, the surface of the NCM811 cathode was clean, allowing the primary particles to be distinguished. Our research revealed that the addition of ISDMS created CEI and SEI in the full cell, and the resulting interfacial layers were mostly conserved during cycling, while effectively alleviating electrolyte decomposition. Electrochemically stable CEI can help suppress the irrevocable phase transformation from layered to rock‐salt caused by cation mixing, resulting from the loss of Li‐ion storage sites in the cathodes.^[^ ^48^ ^]^ Comparative XRD analyses were conducted on NCM811 cathodes subjected to 500 cycles with and without the ISDMS additive to address this issue. NCM811 cathodes cycled with the baseline and VC electrolytes resulted in the shifting of the (003) and (104) peaks in the XRD patterns toward the left and right, respectively, compared with the pristine NCM811 cathode (Figure S50a,b, Supporting Information). Notably, this phenomenon was mitigated for the cathode with the ISDMS electrolyte. Moreover, the NCM811 cathode with the ISDMS electrolyte exhibits a higher intensity ratio of (003)/(104), suggesting a reduced phase transition behavior (Figure S50d, Supporting Information). In addition, severe peak splitting between the (108) and (110) peaks in the XRD patterns of cathodes with the baseline and VC electrolytes indicates distortion in the hexagonal structure of the layered phase (Figure S50c, Supporting Information).^[^ ^49^ ^]^ A cross‐sectional SEM analysis demonstrated that ISDMS improved the structure stability of the NCM811 cathodes. Microcracking of the NCM811 cathodes effectively suppressed through ISDMS electrolyte (Figure S51, Supporting Information). The intergranular cracking of the NCM811 cathodes arises from the inhomogeneous delithiation and lithiation levels between primary particles, caused by the formation of a nonuniform CEI. The formation of a nonuniform, thick CEI and the accumulation of insoluble electrolyte decomposition byproducts led to an increase in the cathode thickness. The ISDMS electrolyte suppressed microcracking and thickness increase of the NCM811 cathodes. Furthermore, scanning transmission electron microscopy (STEM) and electron energy loss spectroscopy (EELS) analysis demonstrated the effect of the ISDMS additive on the suppression of the phase transition in the NCM811 cathode at the atomic level. After 500 cycles at 25 °C, the NCM811 cathode with the baseline electrolyte had a thick rock‐salt phase with a size >10 nm. The VC electrolyte showed reduced thickness of the rock‐salt phase to less than 5 nm, whereas the ISDMS electrolyte significantly reduced the thickness of the rock‐salt phase to less than 2.5 nm (**Figure** **7**a–c). The dQ/dV plots for each cycle supported the beneficial role of the ISDMS on inhibiting the phase transition of the NCM811 cathode. As the cycle progressed in the baseline electrolyte, the intensity of the H1‐M transition peak significantly decreased due to the Jahn–Teller distortion of the Ni^3+^ ions and shifted to a higher potential (Figure S52a, Supporting Information).^[^ ^50^, ^51^, ^52^, ^53^ ^]^ This change increased the level of cation mixing during cycling, leading to a more significant phase transition from layered to rock salt. Consequently, the electrochemical reversibility of the NCM811 cathode worsened, resulting in a capacity decline of the NCM811/graphite full cell along with severe deterioration of the NCM811 cathode. However, the intensity of the H1‐M transition peak was maintained, indicating the alleviation of cation mixing in the NCM811 cathodes in the VC and ISDMS electrolytes (Figure S52b,c, Supporting Information). The EELS analysis helped distinguish the electronic structures of the NCM811 cathodes to determine the valence states of the transition metals (Ni and Co ions), providing evidence for the phase transition of the NCM811 cathode. The NCM811 cathode with the baseline electrolyte began to exhibit an O K pre‐edge peak (528 eV) at 13 nm below the surface, whereas the O K pre‐edge peak for the cathode cycled with the ISDMS electrolyte appeared at a depth of 5 nm from the surface (Figure 7d–f). Moreover, this pre‐edge peak had shifted to a higher energy loss compared to the ISDMS electrolyte in the baseline electrolyte. The ISDMS electrolyte effectively inhibited oxygen loss caused by the movement of electrons from the O 1s orbital to a hybrid state comprising O 2p orbital and the 3d orbital of a transition metal atom.^[^ ^54^ ^]^ Moreover, the disparity between the main‐edge peak and pre‐edge peak indicates a change in the energy loss (*ΔE*), which is one of the important indices to confirm the variation in the valence state of transition metals.^[^ ^55^ ^]^ A lower *ΔE* in the NCM811 cathode with the ISDMS electrolyte implies that the unwanted decrease in the valence state of Ni^4+^ to Ni^2+^ or Ni^3+^ on the cathode surface was alleviated^[^ ^56^ ^]^ (Figure 7d–f). Therefore, we confirmed that the ISDMS electrolyte inhibited the structural collapse of the NCM811 cathode by constructing a stable CEI.

## 3 Conclusion

Electrolyte additive‐assisted interfacial engineering is key to creating electrochemically and thermal robust SEI and CEI for high‐performance batteries. In this study, we demonstrated the beneficial roles of a synthetic additive, namely ISDMS, exhibiting nonbonding electron‐rich sites and thermally resistant sulfur atoms on ion‐permeable and heat‐resistant interfacial layers, in improving the fast‐charging and high‐temperature characteristics of NCM811/graphite full cells. Oxygen atoms with nonbonding electrons in the ISDMS structure contributed to forming complexes with Li^+^ ions competing with EC solvent molecules and enabled PF_6_^−^ anions to enter the solvation structure, leading to the construction of a fast‐charging‐adoptable ion‐conductive SEI on the graphite anode. Furthermore, the ISDMS additive created a sulfur‐rich CEI that can endure high‐temperature environments to protect the NCM811 cathode, with less transition metal dissolution and minimized phase transformation. This approach, which regulates electrolyte–electrode interactions and incorporates polar motifs into the interfacial layers, can be applied to advance the design of electrolyte additives and the interfacial engineering of electrodes for the fast‐charging performance and high‐temperature adaptability of rechargeable batteries.

## 4 Experimental Section

### Synthesis of ISDMS

Isosorbide (50 g, 0.34 mol) and methanesulfonyl chloride (78.4 g, 0.684 mol) were added to acetonitrile (250 mL) in an ice‐salt water bath. When completely dissolved, triethylamine (69.2 g, 0.684 mol, 2 equiv.) was added dropwise over 60 min. The solution blend was agitated at 25 °C for 1 h, and the solid was purified to remove salts. The mixture was recrystallized from isopropyl alcohol (80 g, 77%). ^1^H NMR (400 MHz, CD_3_CN‐d_3_), δ 3.10 (6H, s,‐SH_3_), 3.83 (1H, d,‐CH_2_), 3.93 (2H, m,‐CH_2_), 4.12 (1H, d,‐CH_2_), 4.65 (1H, m,‐CH), 4.87 (1H, m,‐CH), and 5.05 (2H, m,‐CH) were adopted. Isosorbide was supplied from Tokyo Chemical Industry Co. Ltd., and other chemicals were obtained from Sigma‐Aldrich. ^1^H NMR multiplicities were expressed using this format: singlet (s), doublet (d), and multiplet (m).

### Electrolyte and Electrode Fabrication

The baseline electrolyte consisted of 1.15 M of lithium hexafluorophosphate (LiPF_6_) in ethylene carbonate (EC)/diethyl carbonate (DEC) (1:3 volume ratio). These chemicals were obtained from Soulbrain Co., Ltd. The VC and ISDMS electrolytes were fabricated by mixing 1 wt. % of VC and 1 wt. % of ISDMS into the baseline electrolyte, respectively. All electrolytes were filtered by CaH_2_ to reduce water. The electrolyte fabrication process was carried out in an argon‐filled gloved box with a concentration of water and oxygen lower than 1.0 ppm. The NMC811 cathode comprised 96 wt. % active material, 2 wt. % poly(vinylidene fluoride) (Solef 5130, Solvay) binder, and 2 wt. % carbon conducting agent. The cathode had 2.58 mAh cm^−2^ with a mass loading of 12.9 mg cm^−2^. The graphite anode consisted of 96 wt. % of the active material, 1.5 wt. % styrene butadiene rubber binder, 1.5 wt. % carboxymethyl cellulose binder, and 1 wt. % carbon conducting agent. The anode showed 2.9 mAh cm^−2^ with a mass loading of 8.14 mg cm^−2^. Before cell assembly, all the electrodes were vacuum dried at 110 °C for 8 h; an 18 µm polyethylene separator (Tonen Chemicals Corp.) with a porosity of 41% was employed.

### Electrochemical Measurements

The electrochemical behaviors of NCM811/graphite full cells were assessed by 2032 coin‐type cell. The formulation of cells was conducted in an argon‐filled glove box with the amount of water and oxygen lower than 1.0 ppm. The full cells were pre‐cycled at a rate of C/10 from 3 to 4.4 V at 25 °C. Following the charging process to 4.4 V, the voltage was kept constant until the current reached to C/50. The standard cycle was performed for C/5 from 3 to 4.4 V at 25 °C three times after pre‐cycling to stabilize the CEI and SEI layers. The rate performance test was conducted by fixing the discharge C‐rate at C/2 and at different charging C‐rates (C/2, 1 C, 2 C, 3 C, 5 C, and 10 C) (time‐cut off) and back to C/2 every three cycles between 3 and 4.4 V. The cycling behaviors of the full cells were assessed at a fixed charging rate of 1 C and fixed discharging rate of C/2 at 25 °C and 45 °C between 3 and 4.4 V. Under fast‐charging conditions, the charging and discharging rates were changed to 3 C and 1 C, respectively. The electrochemical performance tests were performed using a battery cycler (WBCS 3000, WonAtech).

### Characterization

XPS (K‐alpha, Thermo Fisher Scientific) and TOF‐SIMS (TOF.SIMS 5, IONTOF, positive mode) were performed under ultra‐high vacuum conditions to analyze the interfacial chemistry of the electrode. The beam sources of XPS and TOF‐SIMS were Al‐Kα (1486.7 eV) and Bi_1_^+^ (25 keV), respectively. XRD (SmartLab, RIGAKU) analysis was conducted on the lithiated graphite anode after fast charging using Cu Kα radiation in the 2*θ* range from 20° to 30°. We performed a surface SEM (SU8230, Hitachi) analysis to explore the surface morphologies of the NCM811 cathode and graphite anode after pre‐cycling and 500 cycles. A cross‐sectional SEM analysis of the NMC811 cathode was progressed after ion‐milling (ArBlade5000, Hitachi) to confirm microcracking between the NCM811 particles. STEM (Titan cubed G2 60–300, FEI) was carried out to confirm the components of the SEI on the graphite anode and examine the degradation of the structure in the NCM811 cathode. Prior to the STEM analysis, NCM811 cathodes and graphite anodes were prepared by focused ion beam (FIB) (Helios NanoLab 450, FEI) with carbon layer coating. ESM imaging was performed under Ar‐flowed environment (99.9%, 15 cm^3^ min^−1^, 25 °C) using atomic force microscopy and CES metal body air holder (Cypher‐ES, Oxford Instruments Asylum Research Inc). Pt‐Ir coated tip (EFM‐20, Nanoworld, Spring constant: 2.8 N m^−1^) was used to detect ESM amplitude signal. AC voltage (1 V, 310–370 kHz) was applied to the ESM tip to measure the electrochemical strain response of the SEI, and scanned the tip at a rate of 0.1 Hz over regions of 2 µm × 2 µm. The cross‐sectional image was observed using SEM (SU8230, Hitachi) in an ultrahigh vacuum. STEM, EELS, and EDS data were collected using TEM (Titan cubed G2 60–300, FEI company), and the sampling was conducted using FIB (Thermo Fisher Scientific).

### Density Functional Theory (DFT) Calculation

Material Studio was used for all calculations in this study. The orbital energy levels (HOMO) of the electrolyte components and electrostatic mapping were identified using the DMol^3^ program.^[^ ^57^, ^58^ ^]^ The orbital energy level was determined using Becke's three‐parameter hybrid model with the Lee–Yang–Parr correlation (B3LYP).^[^ ^59^, ^60^ ^]^ All‐electron relativistic core treatments and a double‐numerical plus polarization basis set (version 4.4) were employed to express core electrons and atomic orbital. For the geometry optimization, the specific parameters for the maximum energy change, force, and the displacement were 2 × 10^−5^ Ha, 0.004 Ha Å^−1^, and 0.005 Å, respectively. The dielectric constant of the solvent environment for EC/DEC (1:3 vol%) at 25 °C calculated using the mixing rule was determined to be 10.67.^[^ ^61^ ^]^ The dielectric constant of the EC and DEC were 95.3 and 2.82,^[^ ^62^ ^]^ respectively.

## Conflict of Interest

The authors declare no conflict of interest.

## Author Contributions

S.K., S.P., N.‐S.C., and D.Y. proposed and designed the project. S.K., S.P., and M.K. performed sample preparation and electrochemical evaluation. S.K. synthesized the electrolyte additive. Y.C., G.K., and S.H. analyzed the ionic conductivity of SEI formed on graphite anodes. S.K. performed DFT calculations. S.K., S.P., and N.‐S.C. wrote the manuscript, and N.‐S.C. supervised this work. All authors discussed the results and commented on the manuscript.

## Supporting information



Supporting Information

## Data Availability

The data that support the findings of this study are available on request from the corresponding author. The data are not publicly available due to privacy or ethical restrictions.
