# Supplementary material for: Improving Fast‐Charging Performance of Lithium‐Ion Batteries through Electrode–Electrolyte Interfacial Engineering
Source: Adv Sci (Weinh). 2024 Nov 22;12(3):2411466. doi: 10.1002/advs.202411466 (PMC11744671; doi:10.1002/advs.202411466)
Supplement: Supplementary file 1 — Supporting Information [file ADVS-12-2411466-s001.docx]

Supporting Information

**Improving Fast-Charging Performance of Lithium-Ion Batteries Through Electrode–Electrolyte Interfacial Engineering**

Seungwon Kim, Sewon Park, Minjee Kim, Yoonhan Cho, Gumin Kang, Sunghyun Ko, Daebong Yoon, Seungbum Hong, Nam-Soon Choi^*^

**Experimental Section**

*DFT Calculations:* All calculations were performed using Materials Studio. To calculate the electrostatic potential mapping, Becke’s three-parameter hybrid exchange functional with the Lee–Yang–Parr correlation functional (B3LYP) was applied.^[1,2]^ Generalized gradient approximation (GGA) with Becke–Lee–Yang–Parr (BLYP)^[3]^ density functional was used for geometry optimization. All-electron relativistic treatments and double-numerical plus polarization (version 4.4 basis set) were used to express core electrons and atomic orbital basis set. The specific parameters used for the geometry optimization are as follows: 2×10^−5^ Ha for the maximum energy change, 0.004 Ha Å^−1^ for the force, and 0.005 Å for the displacement. The dielectric constant of the solvent environment for EC/DEC (1:3 vol%) at 25 ℃ was determined to be 10.67, calculated using the mixing rule.^[4]^ The binding energy (${\Delta E}_{\mathrm{binding}}$) between the Li^+^ ion and EC molecule or ISMDS molecule was calculated as follows:

${\Delta E}_{\mathrm{binding}}={E_{\mathrm{Li}^{+}-X}-(E}_{\mathrm{Li}^{+}}+E_{X}) (X=EC or ISDMS)$, (1)

where $E_{\mathrm{Li}^{+}-X}$, $E_{\mathrm{Li}^{+}}$, and $E_{X}$ represent the total energy values of the Li^+^ ion-adsorbed EC or ISDMS, bare Li^+^ ion, and isolated EC or ISDMS molecule, respectively.

*Molecular Dynamics (MD) Simulations:* MD simulations were conducted to investigate the participation of the ISDMS additive in the solvation structure. The COMPASS III forcefield was used to describe the solvation model with EC, DEC, LiPF_6_, and ISDMS molecules.^[5]^ Van der Waals interactions were accounted for by adopting an atom-based cut-off distance of 15.5 Å, whereas electrostatic interactions were analyzed using the Ewald method.^[6]^ The following conditions were applied to optimize the amorphous cell. The maximum energy change was set to 0.0001 kcal mol^−1^, and the maximum force was set to 0.005 kcal mol^–1^ Å^–1^; the stress was 0.005 GPa, and the displacement was 5×10^−5^ Å. The constant volume and energy (NVE) simulation was performed for 10 ps with a time step of 1 fs at 298 K and 1 atm.

*Electrochemical Evaluations:* Linear sweep voltammetry evaluation was performed using VSP-300 (Biologic) with a Li/stainless steel cell at a scan rate of 1 mV s^−1^ from OCV to 5.5 V vs Li/Li^+^ at 25 ℃. The amount of leakage current was measured while maintaining the voltage for 10 h after charging to 4.4, 4.5, and 4.6 V vs Li/Li^+^ at a rate of C/5 at 25 ℃, after the standard cycle of the NCM811/graphite full cell. Electrochemical impedance spectroscopy of the full cells was conducted using an AC impedance analysis with VSP300 (BioLogic) at frequencies ranging from 0.01 to 1 MHz. The NCM811/graphite full cell was charged at 25 ℃ at rates of 3, 5, and 10 C (time-cut off) until 4.4 V vs Li/Li^+^ to investigate Li plating on the graphite anode under fast charging. Electrochemical charging was conducted at 4.4 V vs Li/Li^+^ at charging rates of 3, 5, and 10 C (time cut-off). The pouch cells were fabricated using NCM811 cathode (with a size of 33 mm × 50 mm and a mass load of 12.9 mg cm^-2^), graphite anode (with a size of 35 mm × 52 mm and a mass load of 8.14 mg cm^-2^), and polyethylene separator (18 μm, 41% of porosity, Tonen Chemicals Corp.) with baseline and ISDMS electrolyte. The E/C ratio of the pouch cell was 10.4 mg mAh^-1^. These pouch cells were pre-cycled at a rate of C/10 from 3 to 4.4 V at 25 °C. Following the charging process to 4.4 V, the voltage was kept constant until the current reached to C/50. The standard cycle was performed for C/5 from 3 to 4.4 V at 25 °C three times after pre-cycling to stabilize the cathode–electrolyte interface (CEI) and solid–electrolyte interphase (SEI) layers. All electrochemical evaluations were performed using a battery cycler (WonATech, WBCS 3000).

*Characterization:* ^1^H nuclear magnetic resonance (NMR; AVANCE 3 HD, Brucker) spectroscopy was conducted at 400 MHz to characterize the ISDMS structure. CD_3_CN-d_3_ (NMR grade, Sigma Aldrich) was used as the internal reference. To investigate the complexing of ISDMS with the LiPF_6_ salt, we conducted ^7^Li, ^19^F, and ^31^P NMR spectroscopy (AVANCE 3 HD, 400 MHz, Bruker) using 1 M of lithium chloride (LiCl, Sigma Aldrich) in D_2_O (Sigma Aldrich), 1 wt. % of trimethyl phosphate (TMP, Sigma Aldrich), or 1 wt. % of hexafluorobenzene (Sigma Aldrich) in tetrahydrofuran-d8 (NMR grade, Eurisotop, Saint Aubin) as the reference solution, respectively. The solvation structures of the baseline and ISDMS-containing electrolytes were investigated by ^7^Li NMR with 1 M LiCl in D_2_O as the reference.

The distribution of the relaxation time (DRT) analysis was performed using the pyDRTtools software to elucidate the impedance properties of NCM811/graphite full cells during fast-charging (3 C) cycle. The relationship between the impedance and relaxation time is expressed as follows:^[7–9]^

$Z\left( \omega\right)=R_{0}+R_{pol}\int_{0}^{\infty} \frac{g(\tau)}{1+j\omega\tau}d\tau$, (2)

where *R*_0_ is the ohmic resistance, *g*(*τ*) is the distribution function of the relaxation time, and *R*_pol_ is the polarization resistance.

Li plating on the graphite anode was conducted through an SEM (SU8230, Hitachi) analysis retrieved from fully-charged NCM811/graphite full cells at charging rates of 3, 5, and 10 C (time-cut off) at 25 ℃. In addition, an SEM analysis was performed to examine the surface morphology of the NCM811 cathode and graphite anode after 500 cycles at 25 ℃. X-ray photoelectron spectroscopy (XPS; K-alpha, Thermo Fisher Scientific) was performed on the NCM811 cathode and graphite anode after pre-cycling and 500 cycles to investigate the CEI and SEI on the NCM811 cathode and graphite anode, respectively. The X-ray source of the XPS equipment was Al-Kα (*hν*= 1486.6 eV) radiation.Raman spectroscopy (ARAMIS, Horiba Jobin Yvon) was performed using a 785 nm laser. A differential scanning calorimetry (DSC1, Mettler Toledo) analysis was performed in an N_2_ (>99.999%) atmosphere. The DSC samples comprised 2:1 wt. % of de-lithiated NCM811 with electrolyte, and the total weight of the sample was 2.1 mg. The de-lithiated NCM811 was prepared by charging after standard cycling of the NCM811/graphite full cells under a rate of C/10 up to 4.4 V at 25 ℃. The samples were heated at temperatures ranging from 30 to 400 ℃ at a rate of 5 ℃ min^−1^. An inductively coupled plasma-optical emission spectrometer (ICP-OES, 700-ES, Varian) was used to measure the amounts of dissolved transition metal ions in electrolytes that were stored in contact with the fully-delithiated NCM811 cathode in a 60 ℃ oven for 3 d. For the ICP analysis, the delithiated NCM811 cathodes were obtained from NCM811/graphite full cells charged up to 4.4 V after pre-cycling at C/10 and 25 ℃. XRD (SmartLab, RIGAKU) with Cu Kα radiation in the 2*θ* range of 10°–80° to examine the deterioration of the NCM811 cathode retrieved from the NCM811/graphite full cells after 500 cycles at 25 ℃.

**Table S1.** Comparison of fast-charging cycle performance between our study and previously reported electrolyte systems in LIB

| Cathode | Anode | Electrolyte | Operation  voltage | Cycle performance | | Ref |
| --- | --- | --- | --- | --- | --- | --- |
|  |  |  |  | Ref. | Exp. |  |
| NCM 811 | Gr | 1.15 M LiPF_6_ in EC/DEC (1:3 vol%) +1% ISDMS | 3–4.4 V | 62.8% @ 3C/1C, 500 cycles | 76.7% @ 3C/1C,  500 cycles | This work |
| NCM 811 | Si-C  (3% of Si) | 1.15 M LiPF_6_ in EC/EMC (3:7 vol%) + 0.5% VC+0.5% DMVC-OCF_3_+0.5% DMVC-OTMS | 2.5–4.3 V | 63.8%  @ 3C/1C,  100 cycles | 98.2%  @ 3C/1C,  100 cycles | [R10] |
| NCM 811 | Gr | 1 M LiPF_6_ in FEC/AN (7/3 vol%) | 3–4.4 V | 12.6%  @ 4C/0.3C, 100 cycles | 74.8%  @ 4C/0.3C,  100 cycles | [R11] |
| NCM 811 | Gr | LiFSI:DMC:EC:TTE  (1:4.8:0.2:1 mol%) | 2.5–4.4 V | 69%  @ 4C/0.2C, 300 cycles | 82.3%  @ 4C/0.2C, 300 cycles | [R12] |
| NCM 622 | Gr | 1.15M LiPF6 in EC:DMC (5:95 vol%)  + 0.1% TMSNCS | 3–4.4 V | 59.9%  @ 2C/1C, 150 cycles | 67.4%  @ 2C/1C,  150 cycles | [R13] |
| LFP | Gr | 1 M LiPF_6_ in EC/DMC  (1:1 vol%)+5% FEC+5% triglyme+5% LiNO_3_ | 2–4.25 V | 30.9%  @ 2C/1C,  200 cycles | 61.2% @ 2C/1C,  200 cycles | [R14] |
| LPF | Gr | 1.6 M LiFSI in EC/DMC/HFE  (1/30/40 vol%) | 2–4.25 V | 30%  @ 2C/1C, 120 cycles | > 80%  @ 2C/1C,  400 cycles | [R15] |

Gr: graphite


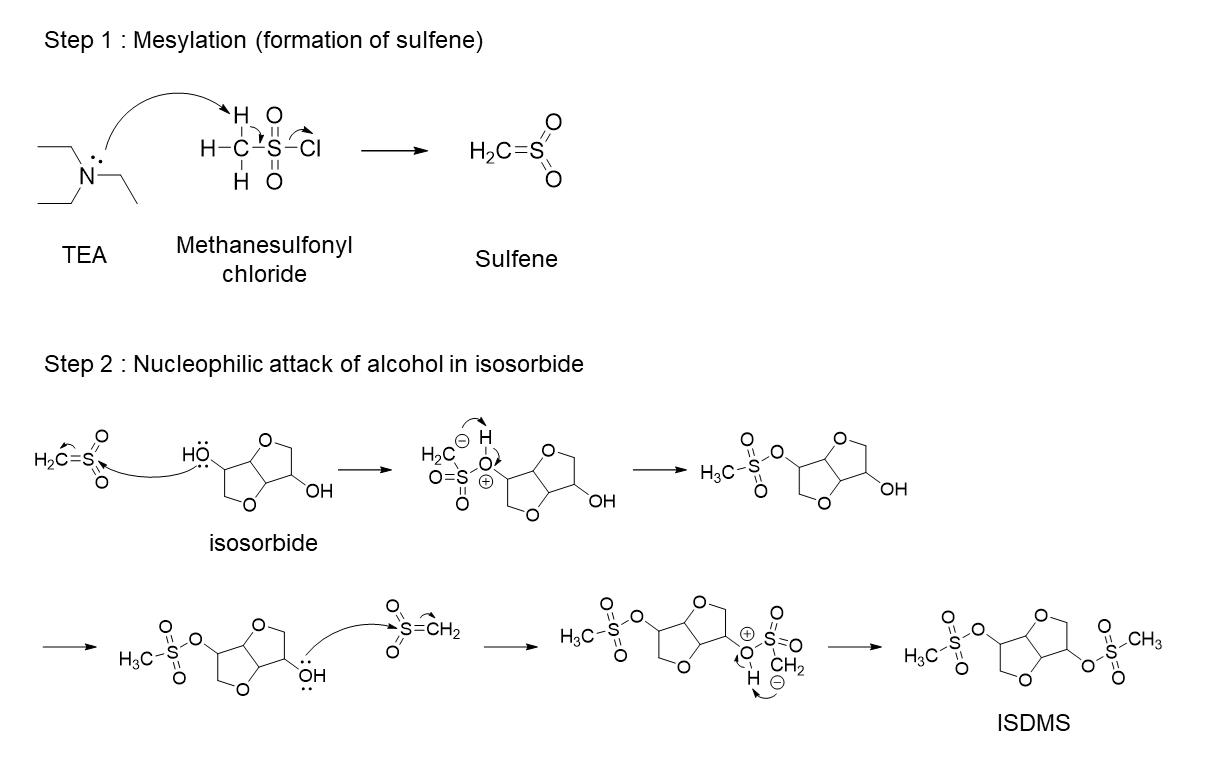


**Figure S1.** Detailed synthetic mechanism of ISDMS.


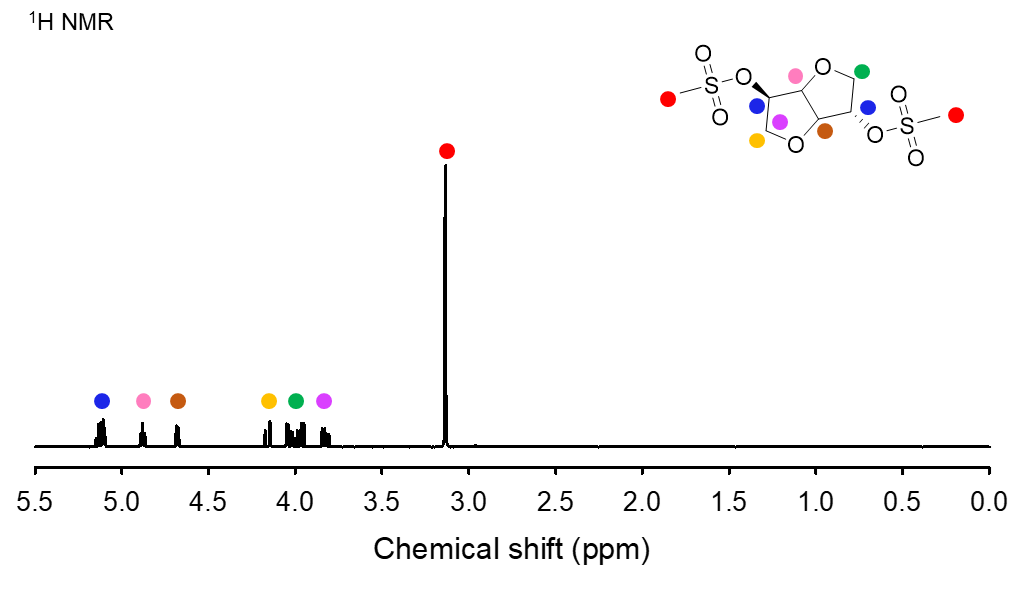


**Figure S2.** ^1^H (400 MHz, CD_3_CN-d_3_) NMR spectra of synthesized ISDMS. δ 3.10 (6H, s, -SH_3_), 3.83 (1H, d, -CH_2_), 3.93 (2H, m, -CH_2_), 4.12 (1H, d, -CH_2_), 4.65 (1H, m, -CH), 4.87 (1H, m, -CH), and 5.05 (2H, m, -CH).


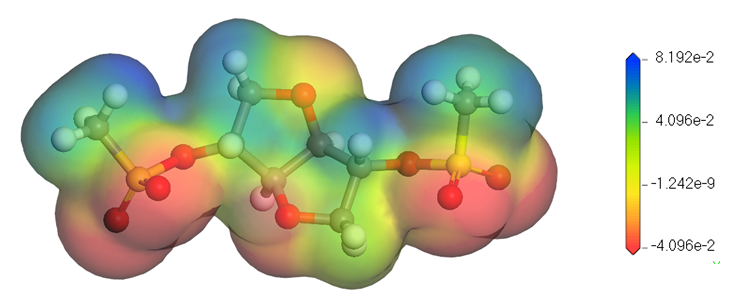


**Figure S3.** Electrostatic potential mapping of ISDMS additive.


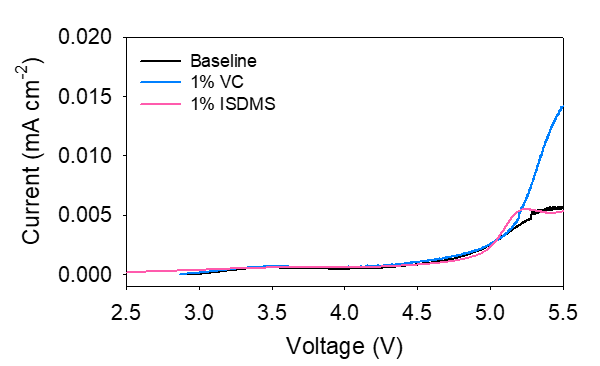


**Figure S4.** Linear sweep voltammetry (LSV) results for Li/stainless steel cells with baseline, 1% VC, and 1% ISDMS electrolytes at a scanning rate of 1 mV s^−1^ at 25 ℃.


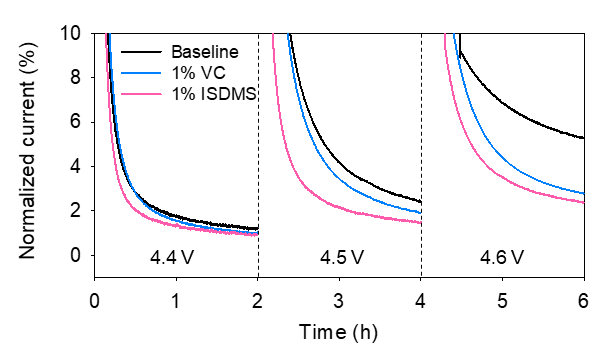


**Figure S5.** Normalized leakage currents of NCM811/Li half cells with baseline, 1% VC, and 1% ISDMS electrolytes under 4.4, 4.5, and 4.6 V vs. Li/Li^+^ at 25 ℃ after pre-cycling.


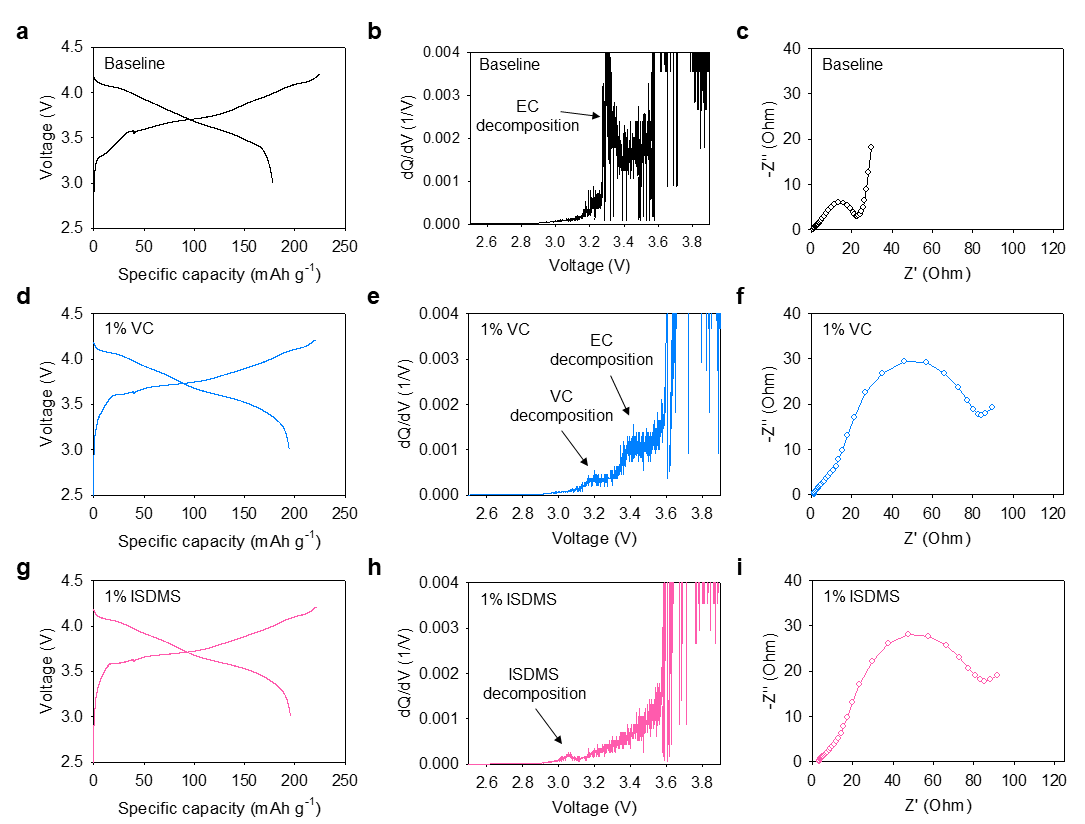


**Figure S6.** a, d, g) Charge/discharge voltage curves, b, e, h) dQ/dV plots, and c, f, i) Nyquist plots of NCM811/graphite full cells during pre-cycling at 25 ℃ with a, b, c) baseline, d, e, f) 1% VC, and g, h, i) 1% ISDMS electrolytes with charge cut-off voltage of 4.4 V at C/10 and 25 ℃.

**
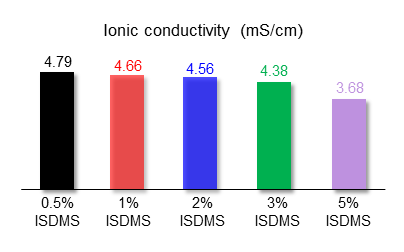
**

**Figure S7.** Ionic conductivity of different concentrations of ISDMS containing electrolytes at 25 ℃.


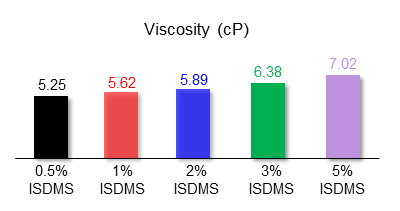


**Figure S8.** Viscosity values for different concentrations of ISDMS containing electrolytes at 25 ℃.


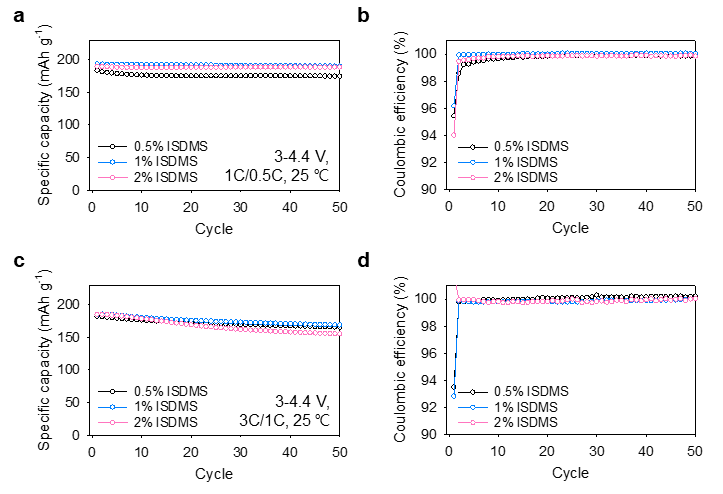


**Figure S9.** a, c) Cycle performance and b, d) Coulombic efficiency of NCM811/graphite full cells with 0.5%, 1%, and 2% ISDMS electrolytes for a, b) 1C/0.5C cycling and c, d) 3C/1C cycling.


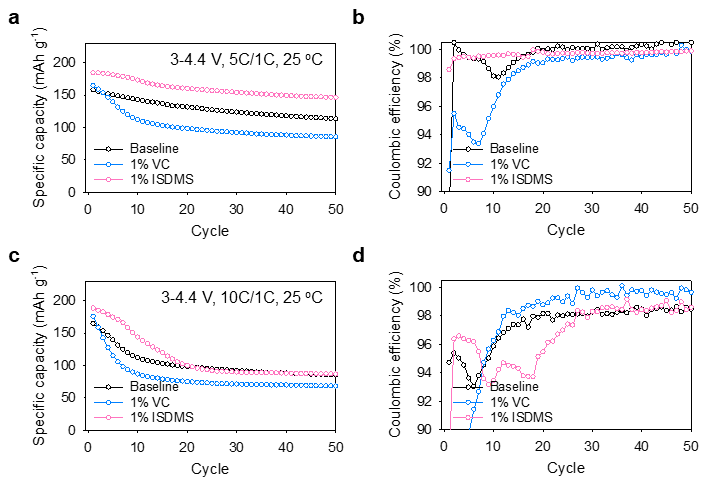


**Figure S10.** a) Cycle performance and b) Coulombic efficiency of NCM811/graphite full cells at a charging rate of 5 C.


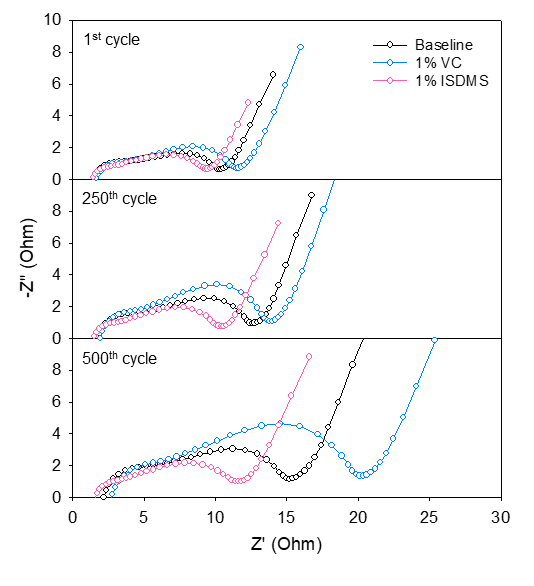


**Figure S11.** Nyquist plots of NCM811/graphite full cells with baseline, 1% VC, and 1% ISDMS electrolytes after 1, 250, and 500 cycles at 25 ℃ under a fast-charging (3 C) condition.


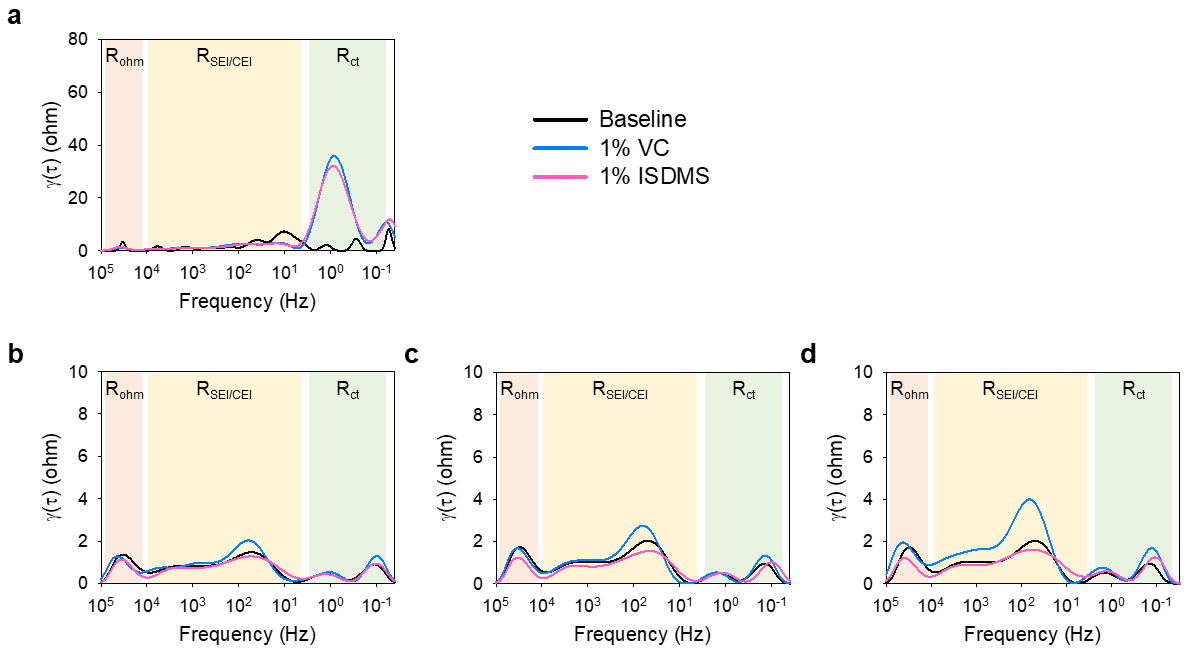


**Figure S12.** DRT analysis of NCM811/graphite full cells after a) pre-cycling, b) 1 cycle, c) 250 cycles, and d) 500 cycles at 25 ℃ under a fast-charging (3 C) condition.


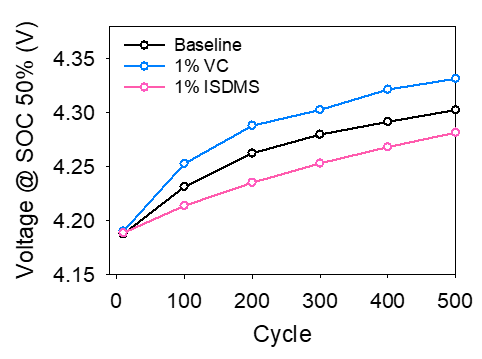


**Figure S13.** Variation in the voltages of NCM811/graphite full cells with different electrolytes in a state of charge (SOC) of 50% for 10, 100, 200, 300, 400, and 500 cycles at 25 ℃ under fast-charging (3 C) conditions.


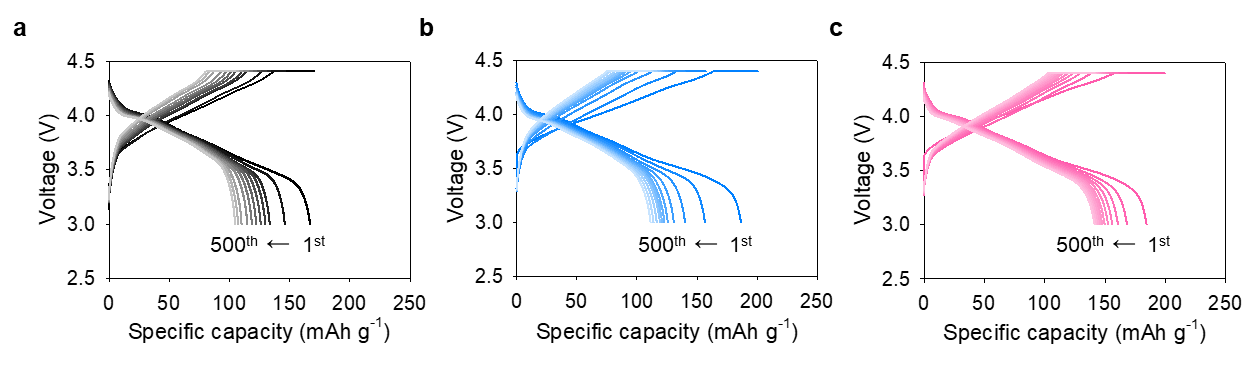


**Figure S14.** Voltage profiles for 1, 50, 100, 150, 200, 250, 300, 350, 400, 450, and 500 cycles of NCM811/graphite full cells for a) baseline, b) 1% VC, and c) 1% ISDMS electrolytes under a fast-charging (3 C) condition at 25 ℃.


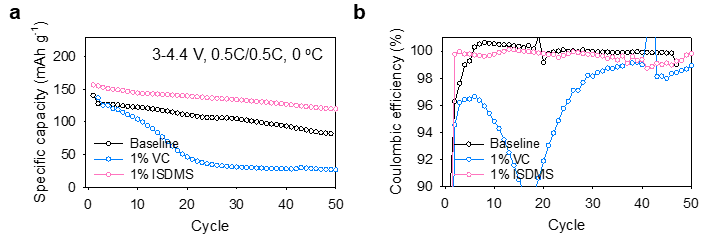


**Figure S15**. a) Cycle performance and b) Coulombic efficiency of NCM811/graphite full cells at 0 ℃.

**
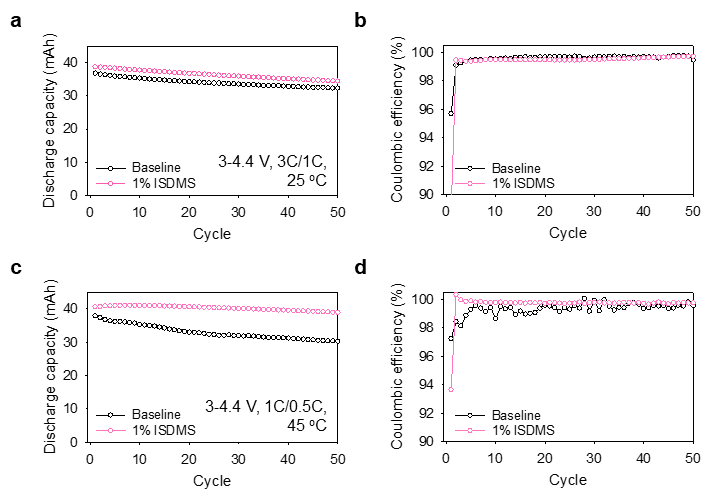
**

**Figure S16.** a, c) Cycle performance and b, d) Coulombic efficiency of NCM811/graphite pouch cells for a, b) fast-charging (3C/1C) cycling and c, d) high-temperature (45 ℃) cycling.


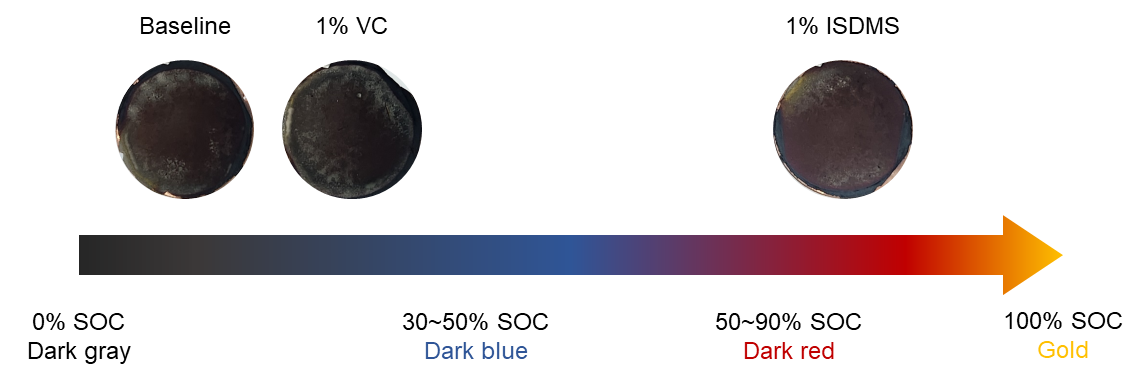


**Figure S17**. Photographs of lithiated (charged) graphite anodes retrieved from NCM811/graphite full cells after 5 C charging at 25 ℃ with baseline, 1% VC, and 1% ISDMS electrolytes (The charging process was conducted by time cut-off at 4.4 V).


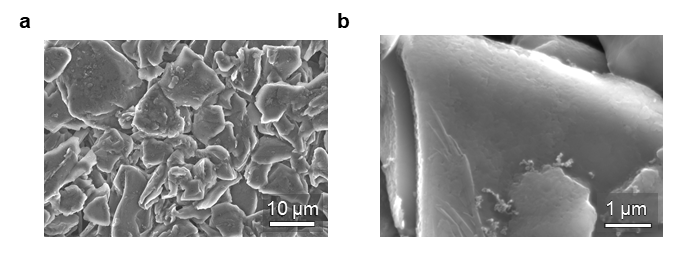


**Figure S18.** Top-view SEM images of pristine graphite anode at a) low and b) high magnitudes.


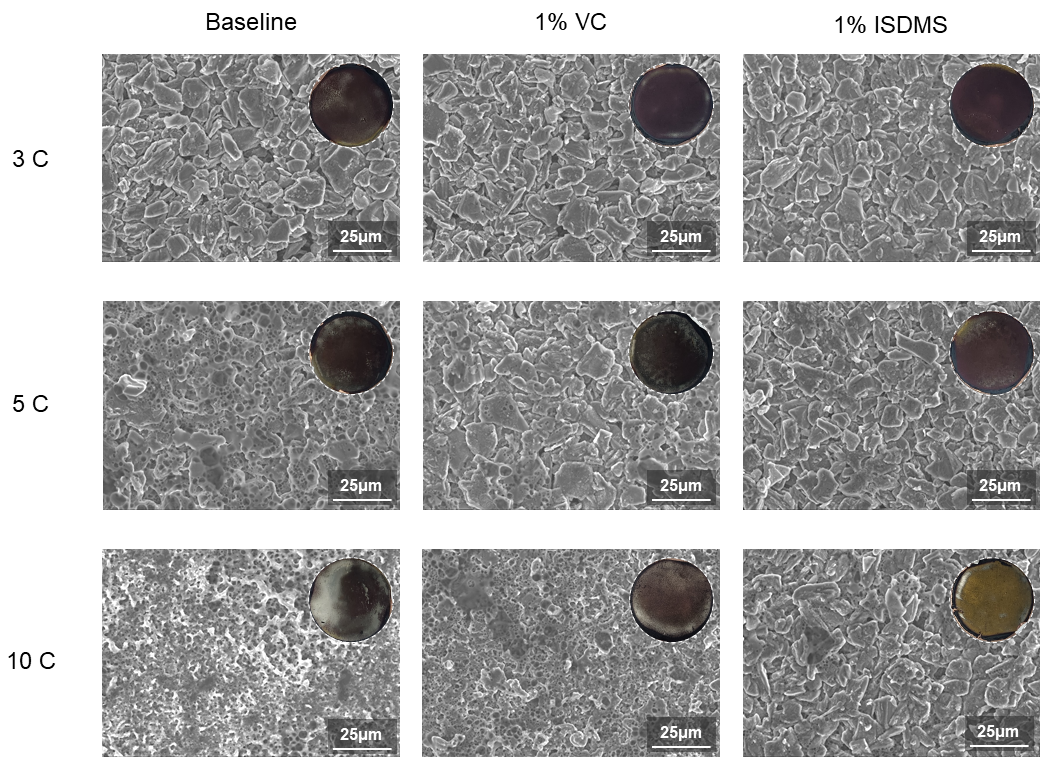


**Figure S19.** Surface SEM images and photographs of lithiated (charged) graphite anodes retrieved from NCM811/graphite full cells after fast (3 C, 5 C, and 10 C) charging at 25 ℃ with baseline, 1% VC, and 1% ISDMS electrolytes. The charging process was conducted by time cut-off (cut-off voltage of 4.4 V).


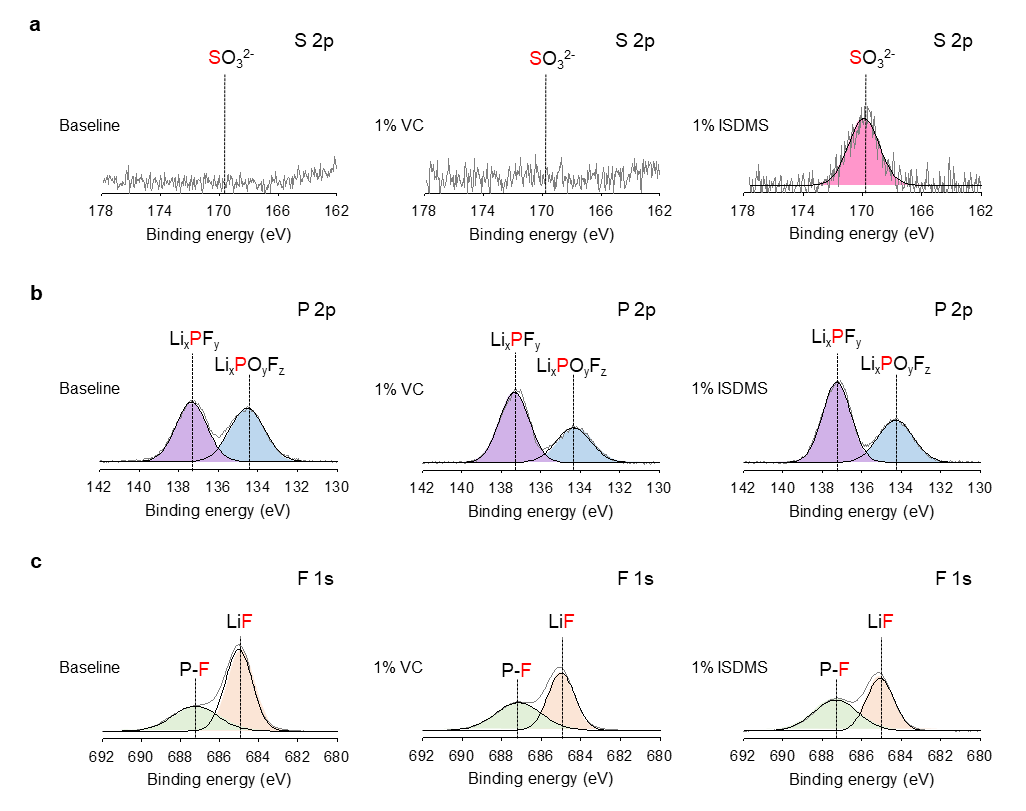


**Figure S20.** a) S 2p, b) P 2p, and c) F 1s XPS of graphite anodes retrieved from NCM811/graphite full cells with baseline, 1% VC, and 1% ISDMS electrolytes after pre-cycling at C/10 and 25 ℃.


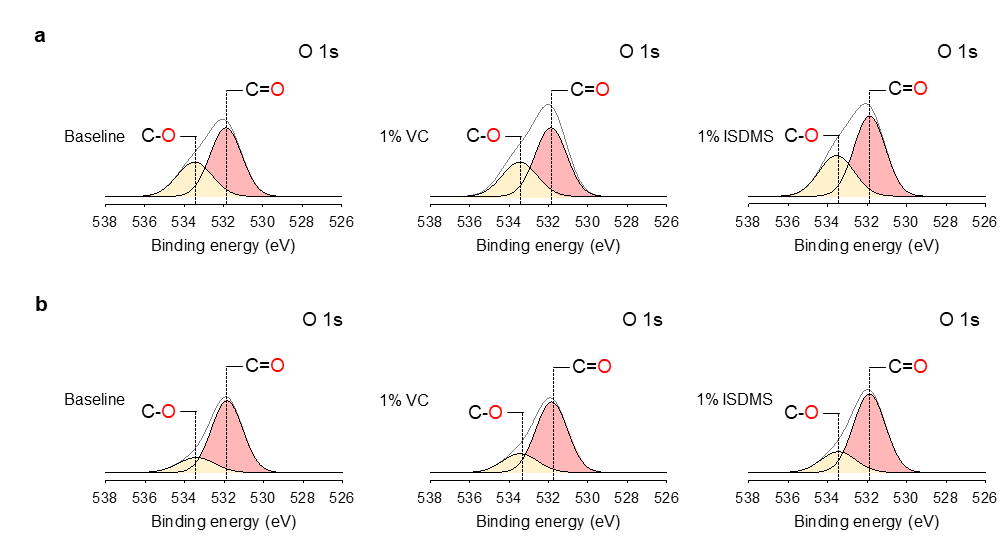


**Figure S21.** O 1s XPS of a) graphite anodes, and b) NCM811 cathodes retrieved from NCM811/graphite full cells with baseline, 1% VC, and 1% ISDMS electrolytes after pre-cycling at C/10 and 25 ℃.


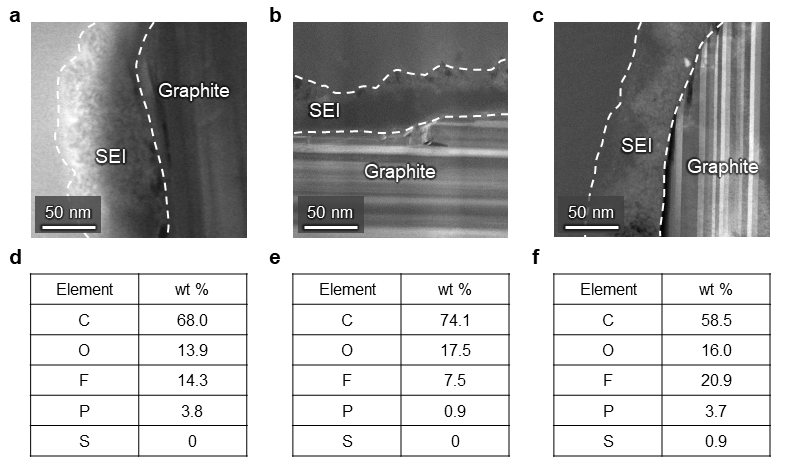


**Figure S22**. TEM images and EDS data of graphite anode with a, d) baseline electrolyte, b, e) 1% VC electrolyte, and c, f) 1% ISDMS electrolyte after pre-cycling.


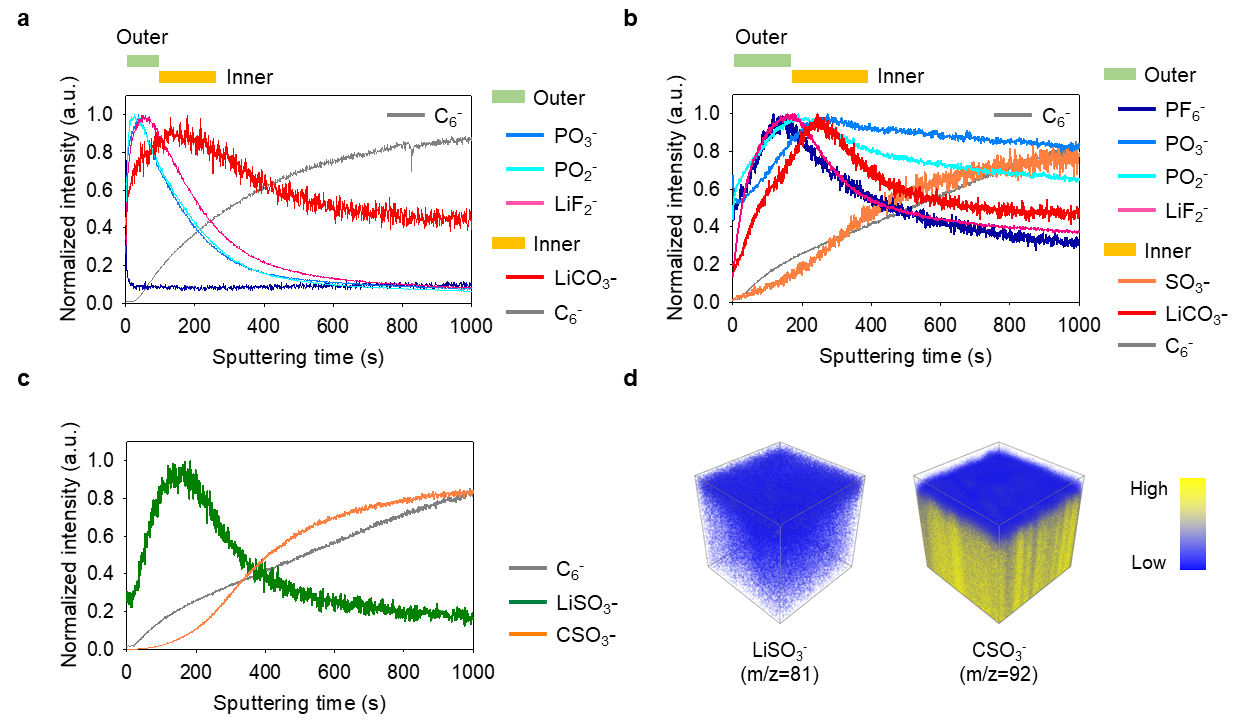


**Figure S23.** TOF-SIMS depth profiles of graphite anodes retrieved from NCM811/graphite full cells with a) baseline electrolyte and b) ISDMS electrolyte after pre-cycling at C/10 and 25 ℃. TOF-SIMS c) depth profile and d) 3D cubic images of graphite anodes retrieved from NCM811/graphite full cells with the ISDMS electrolyte after pre-cycling at C/10 and 25 ℃.


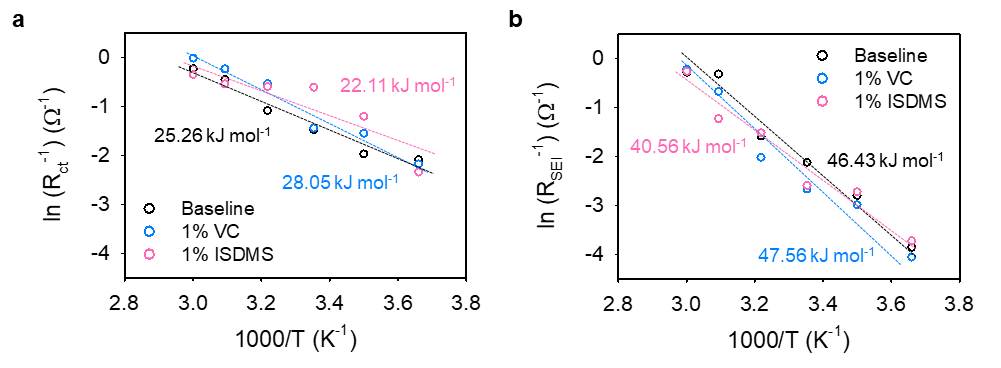


**Figure S24.** a) Desolvation energy of Li^+^ and b) Energy of Li^+^ transport through SEI of electrolytes.


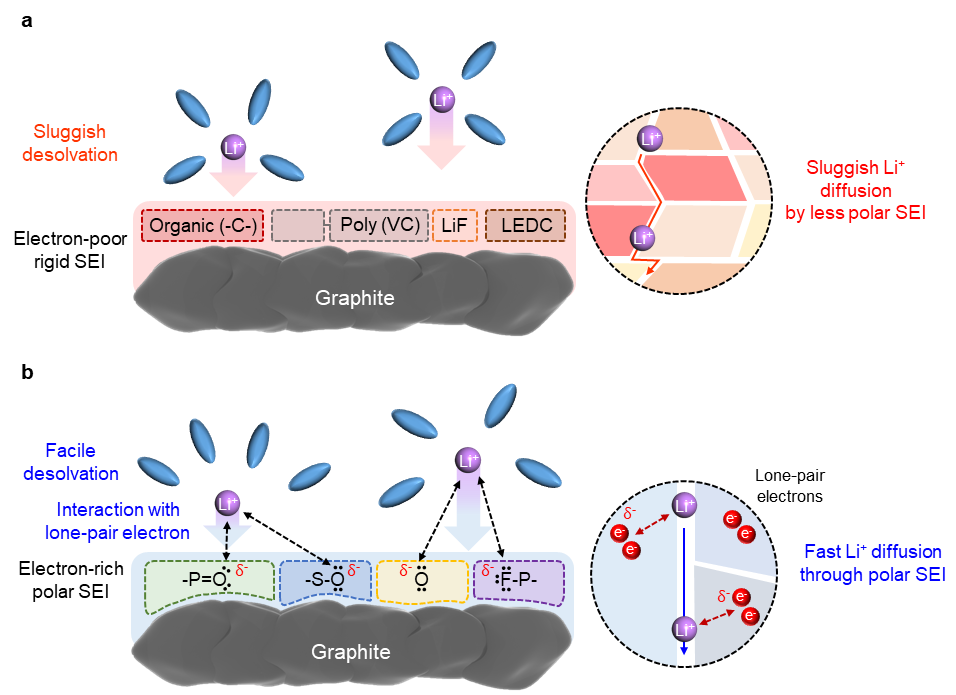


**Figure S25.** Schematic of SEI effect formed by a) baseline and VC electrolytes and b) ISDMS electrolyte.


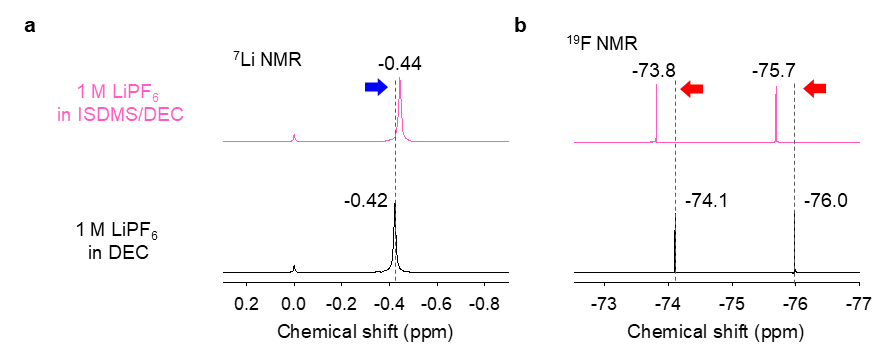


**Figure S26.** a) ^7^Li and b) ^19^F spectra of 1 M of LiPF_6_ dissolved in DEC and 1 M of LiPF_6_ dissolved in ISDMS/DEC (1:9 wt. %).


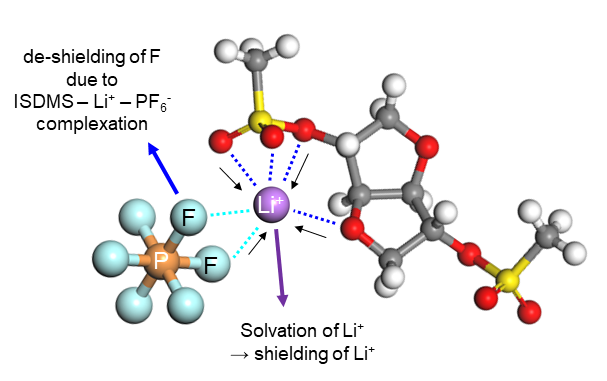


**Figure S27.** Schematic of complexation with ISDMS and LiPF_6_.


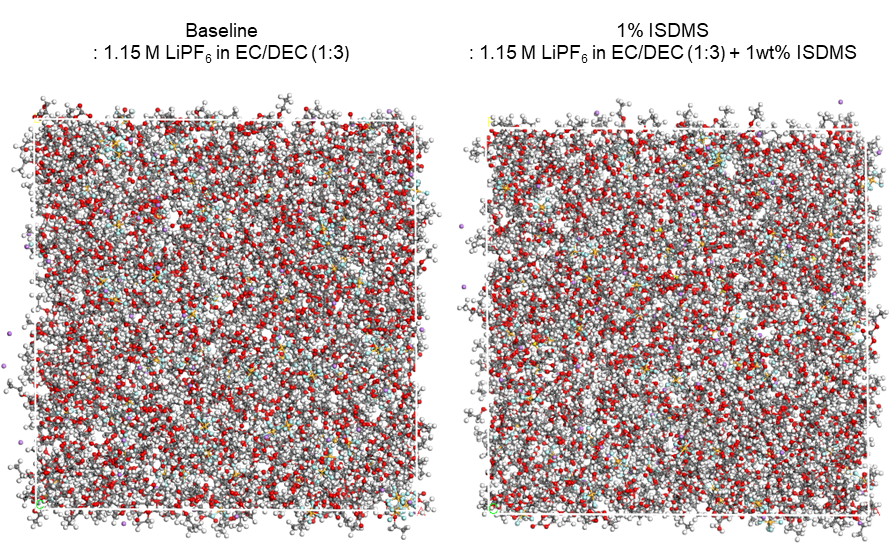


**Figure S28.** Model systems for molecular dynamics (MD) simulation for baseline and 1% ISDMS electrolyte system. For baseline, EC, DEC, and LiPF_6_ molecules are 392, 584, and 108, respectively. For 1% ISDMS, EC, DEC, LiPF_6_, and ISDMS molecules are 392, 584, 108, and 4, respectively.


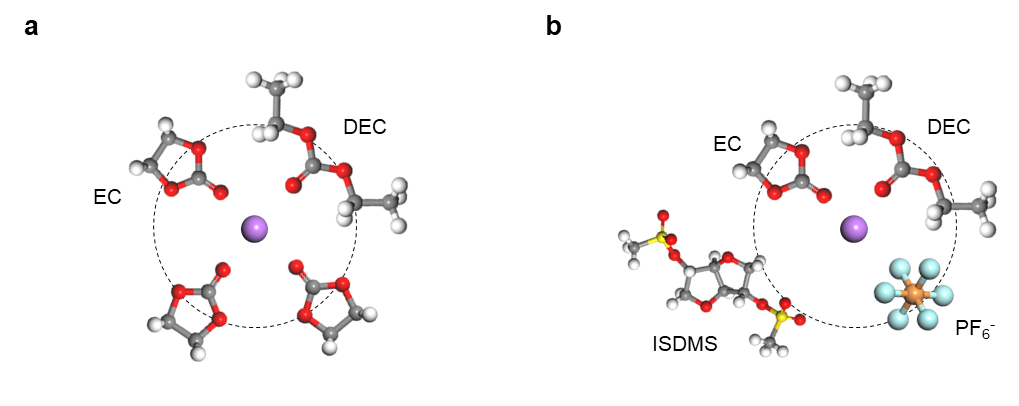


**Figure S29.** Model systems of MD simulation within 5 Å based on Li^+^ and expected solvation structure of a) baseline electrolyte and b) 1% ISDMS electrolyte.


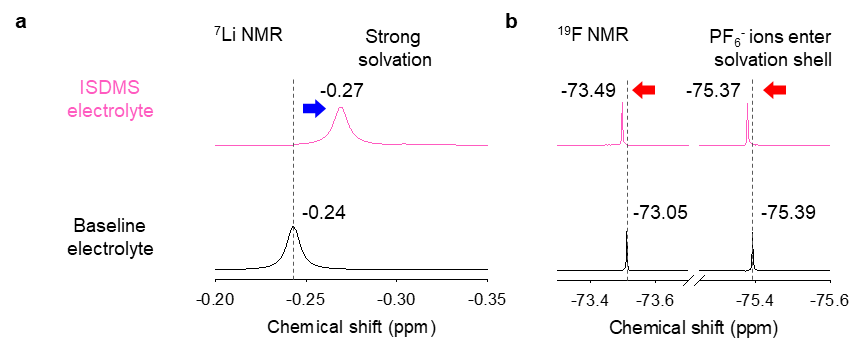


**Figure S30.** a) ^7^Li and b) ^19^F NMR spectra of baseline and ISDMS electrolytes.


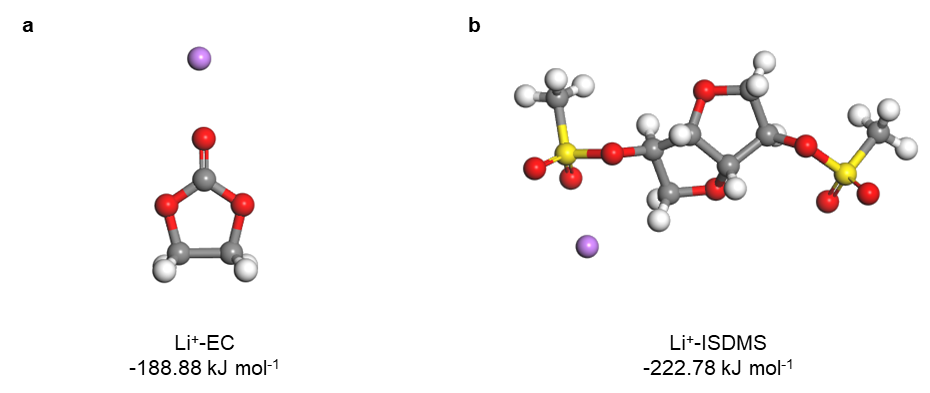


**Figure S31.** Binding energies of a) Li^+^-EC and b) Li^+^-ISDMS.


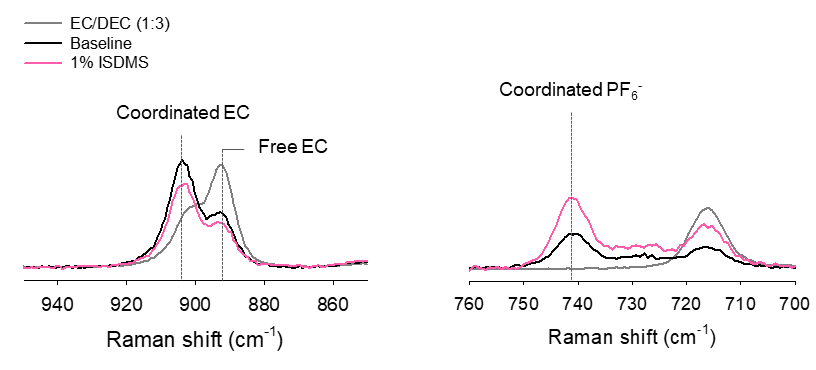


**Figure S32.** Raman spectra for EC/DEC (1:3) mixed solvent, baseline, and 1% ISDMS electrolytes.


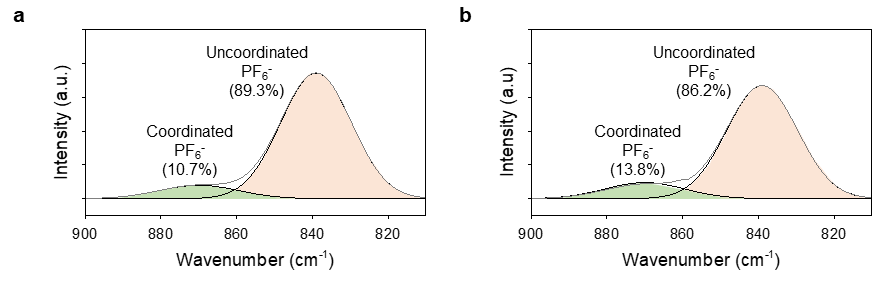


**Figure S33.** FT-IR spectra of the P-F stretching bond of a) baseline electrolyte and b) ISDMS electrolyte.


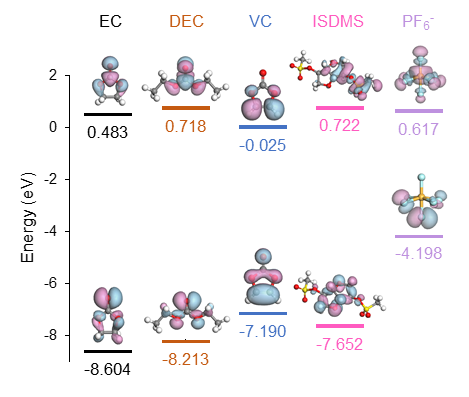


**Figure S34.** HOMO and LUMO energy levels of solvents, ISDMS additive, and PF_6_^-^ anion


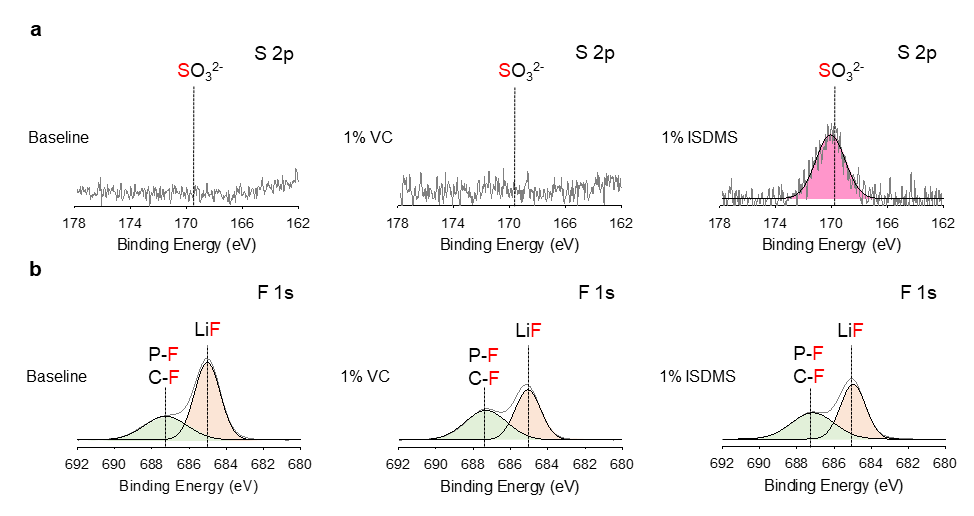


**Figure S35.** a) S 2p and b) F 1s XPS spectra of the NCM811 cathodes after pre-cycling at 25 ℃ retrieved from NCM811/graphite full cells with baseline, 1% VC, and 1% ISDMS electrolytes.


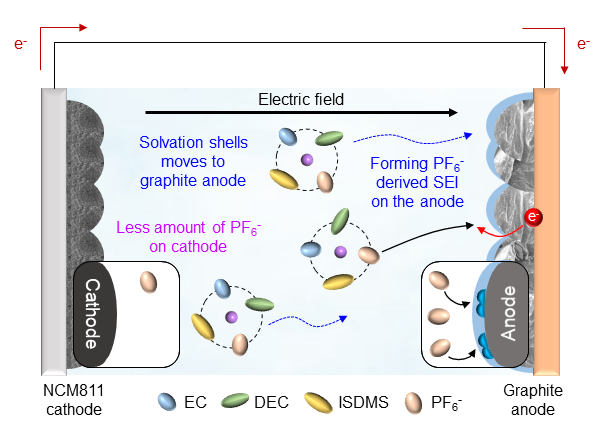


**Figure S36.** Schematic of SEI formation on graphite anode during the initial charging process.


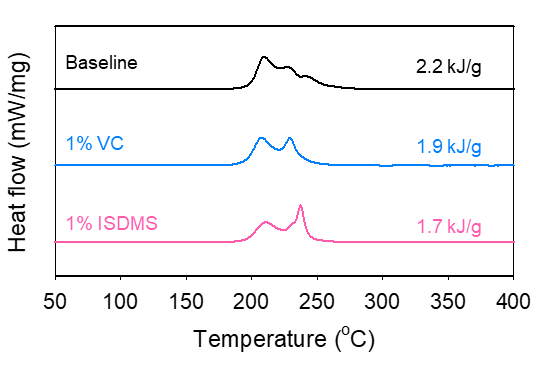


**Figure S37.** DSC heating curves of fully delithiated NCM811 cathodes with baseline (black), 1% VC (blue), and 1% ISDMS (pink) electrolytes. Delithiated cathodes were obtained from NCM811/graphite full cells charged up to 4.4 V at C/10 after pre-cycling at 25 ℃ and a charge cut-off voltage of 4.4 V.


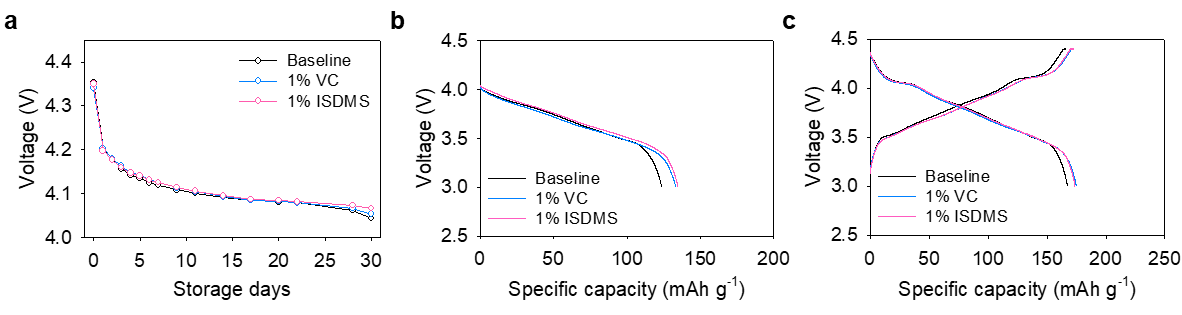


**Figure S38.** a) Open-circuit voltage drop of NCM811/graphite full cells charged up to 4.4 V during storage at 60 ℃. b) Discharge voltage profiles of NCM811/graphite full cells at C/10 after storage for 30 d at 60 ℃. c) Recovery voltage profiles at C/10 after storage for 30 d at 60 ℃.

**
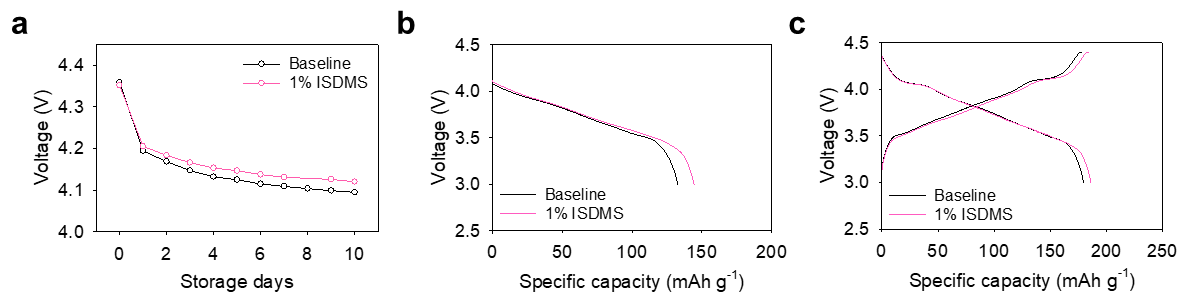
**

**Figure S39.** a) Open-circuit voltage drop of NCM811/graphite pouch cells (42.9 mAh) charged up to 4.4 V during storage at 60 ℃. b) Discharge voltage profiles of NCM811/graphite pouch cells at C/10 after storage for 10 d at 60 ℃. c) Recovery voltage profiles at C/10 after storage for 10 d at 60 ℃.


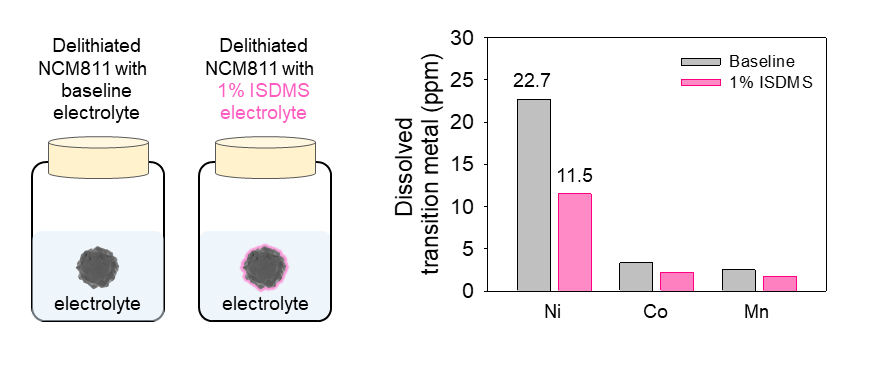


**Figure S40.** ICP-OES results of dissolved contents of transition metal ions from delithiated NCM811 cathodes. Delithiated cathodes charged up to 4.4 V in full cells were stored in the baseline electrolyte for 3 d at 60 ℃.


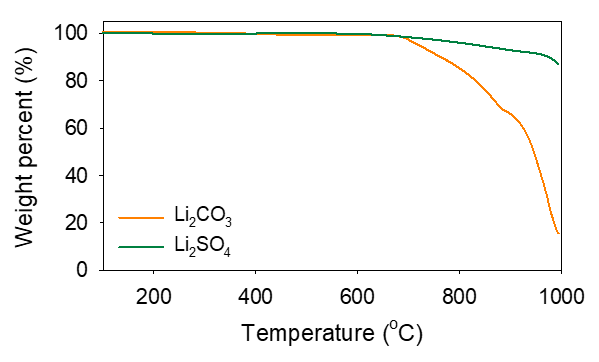


**Figure S41.** TGA results for Li_2_CO_3_ (orange) and Li_2_SO_4_ (green).


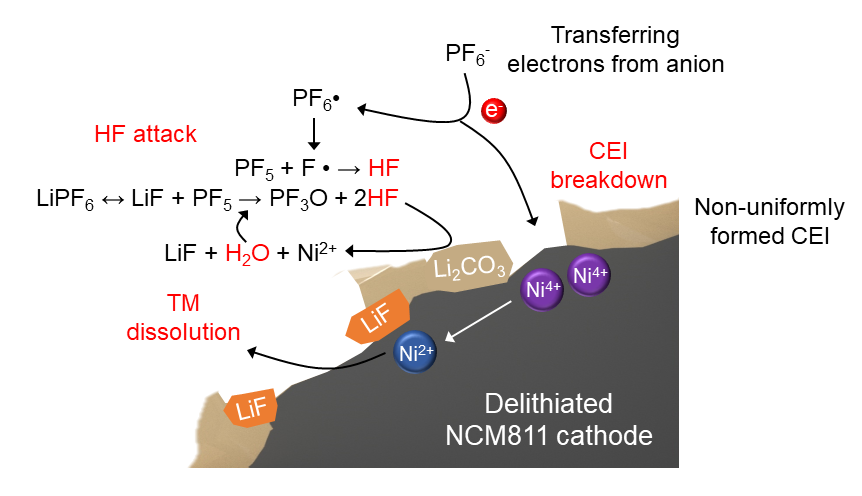


**Figure S42.** Schematic for Ni dissolution out of a delithiated NCM811 cathode via HF attack and anion oxidation.


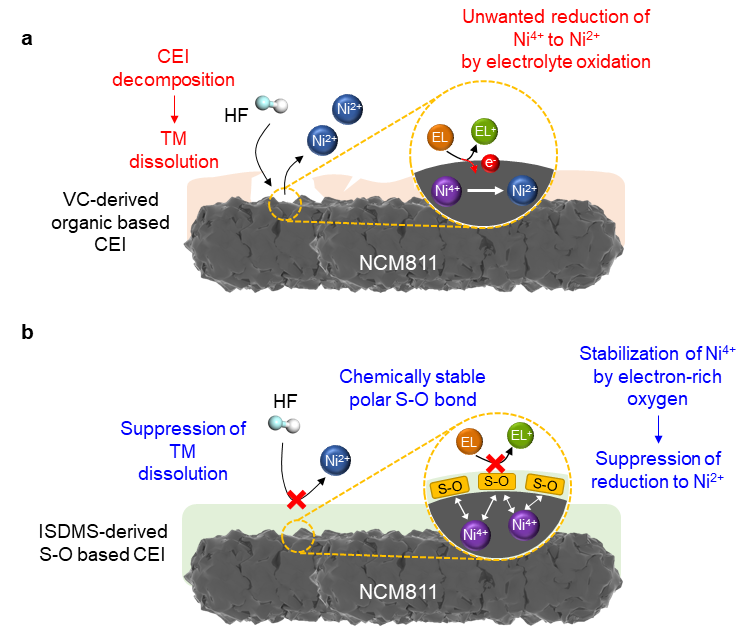


**Figure S43.** Schematic showing different CEI qualities formed by a) baseline and VC electrolytes and b) ISDMS electrolyte.


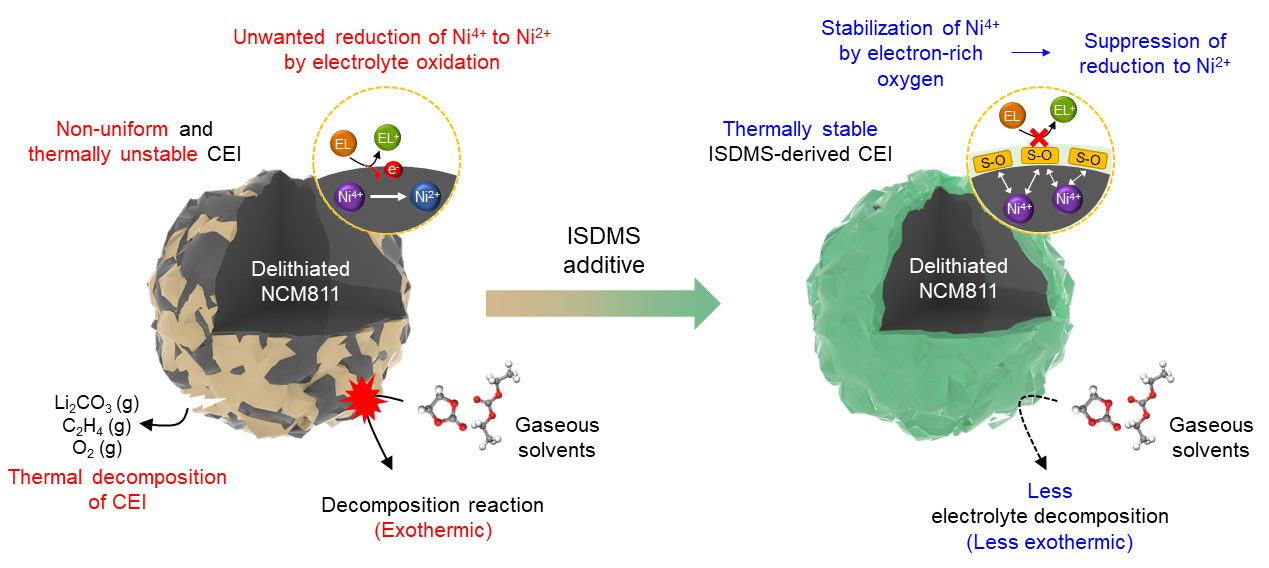


**Figure S44.** Schematic of the function of ISDMS-derived CEI at elevated temperatures.


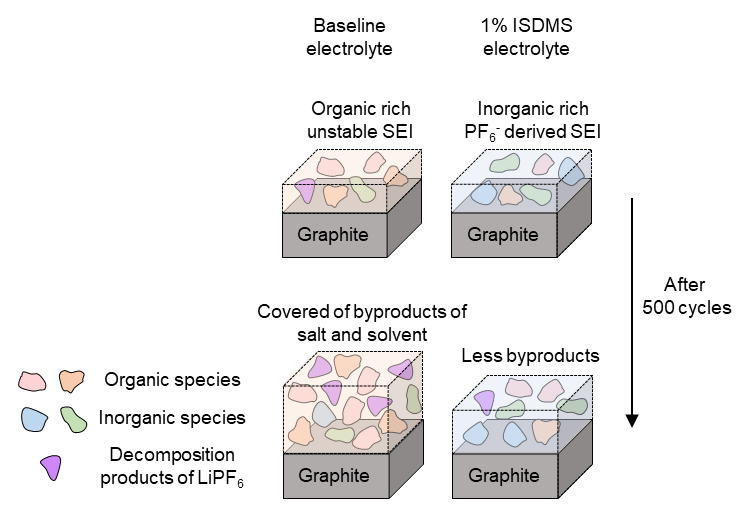


**Figure S45.** Schematic of change in SEI derived by baseline and ISDMS electrolytes as the cycle progressed.


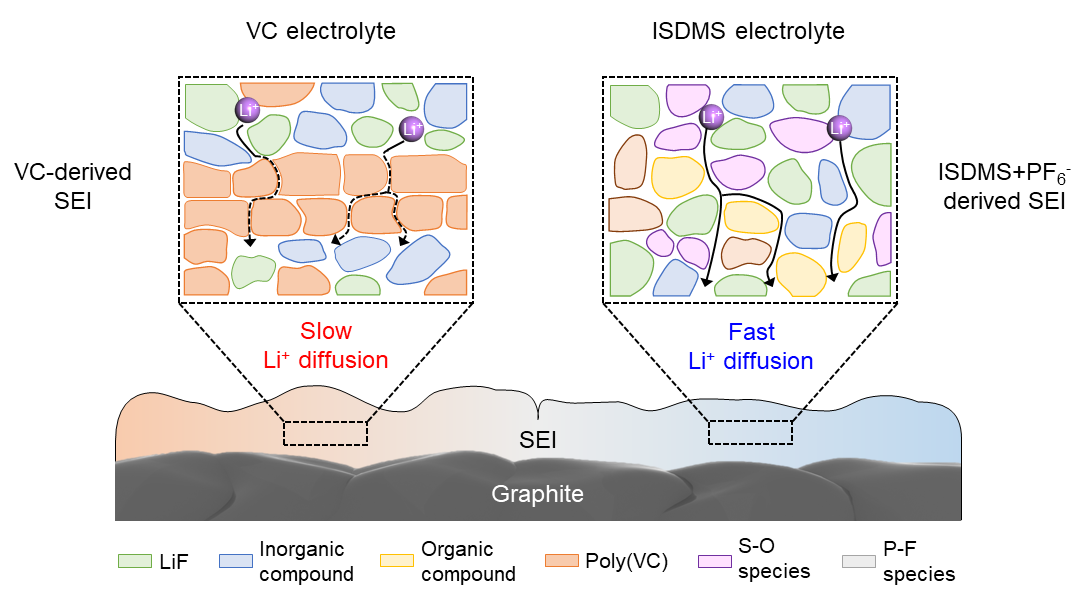


**Figure S46.** Schematic of Li^+^ diffusion in SEI derived by baseline and ISDMS electrolytes.


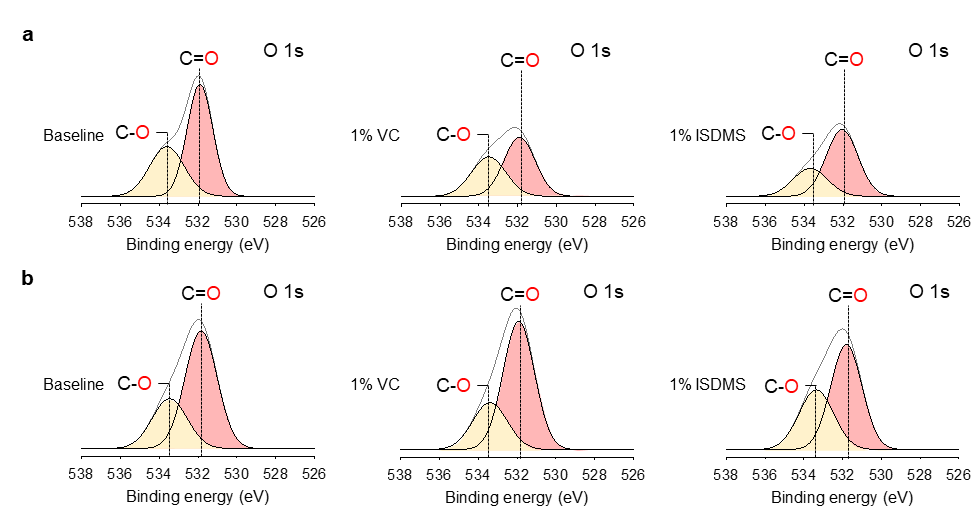


**Figure S47.** O 1s XPS results of a) graphite anodes, and b) NCM811 cathodes extracted from NCM811/graphite full cells with baseline, 1% VC, and 1% ISDMS electrolytes after 500 cycles at 1 C/0.5 C and 25 ℃.


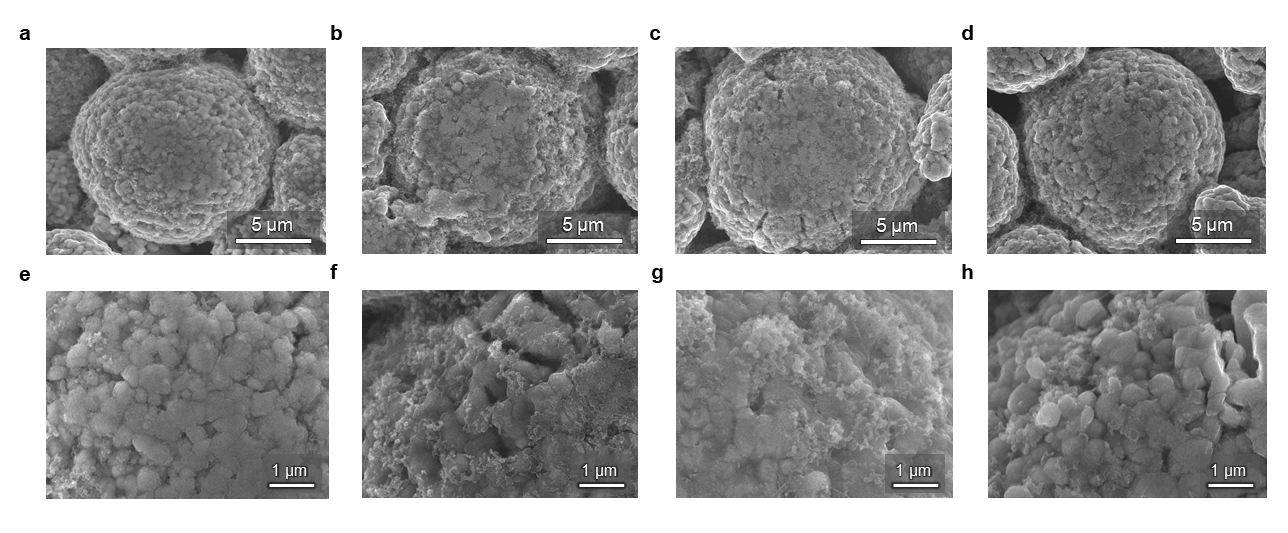


**Figure S48.** Surface morphologies of a, e) pristine NCM811 cathode, NCM811 cathodes retrieved from NCM811/graphite full cells with b, f) baseline, c, g) 1% VC, and d, h) 1% ISDMS electrolytes after 500 cycles at 1 C/0.5 C and 25 ℃.


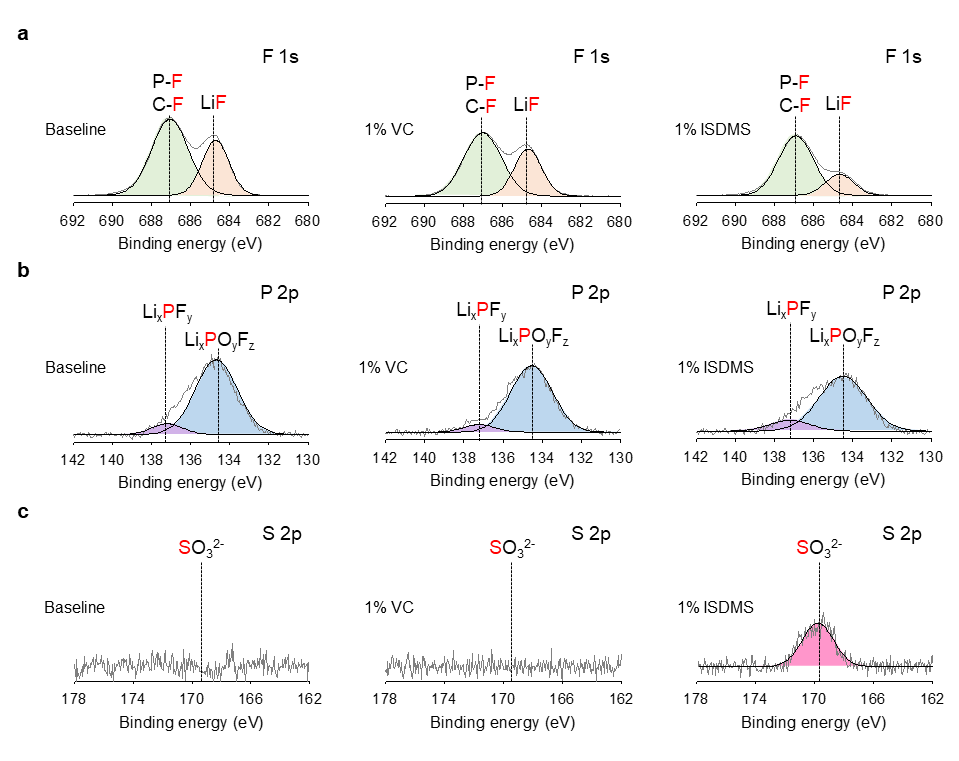


**Figure S49.** a) F 1s, b) P 2p, and c) S 2p XPS results of NCM811 cathodes retrieved from NCM811/graphite full cells with baseline, 1% VC, and 1% ISDMS electrolytes after 500 cycles at 1 C/0.5 C and 25 ℃.


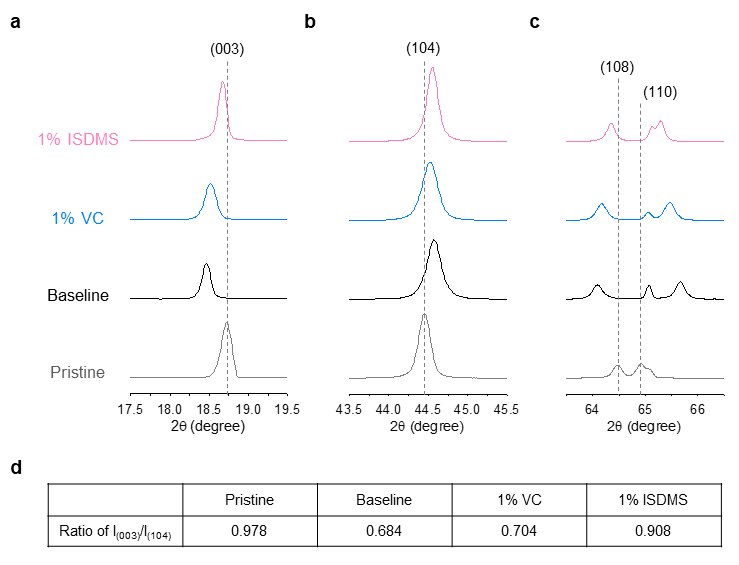


**Figure S50.** XRD patterns of NCM811 cathodes retrieved from NCM811/graphite full cells after 500 cycles at 1 C/0.5 C and 25 ℃ with baseline, 1% VC, and 1% ISDMS electrolytes: a) (003) peak, b) (104) peak, and c) (108), (110) peak. d) Intensity ratio of (003)/(104) peak for each electrolyte.


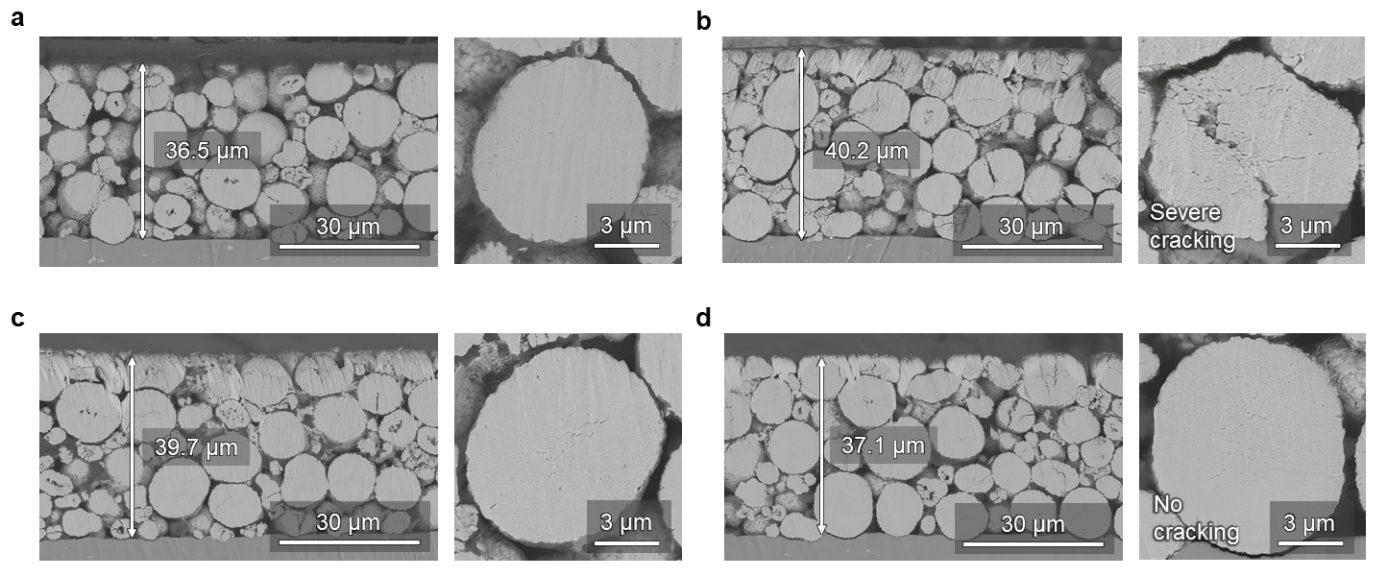


**Figure S51.** Cross-sectional morphologies and microstructures of a) pristine NCM811 cathode, NCM811 cathodes retrieved from NCM811/graphite full cells after 500 cycles at 1 C/0.5 C and 25 ℃ with b) baseline, c) 1% VC, and d) 1% ISDMS electrolytes.


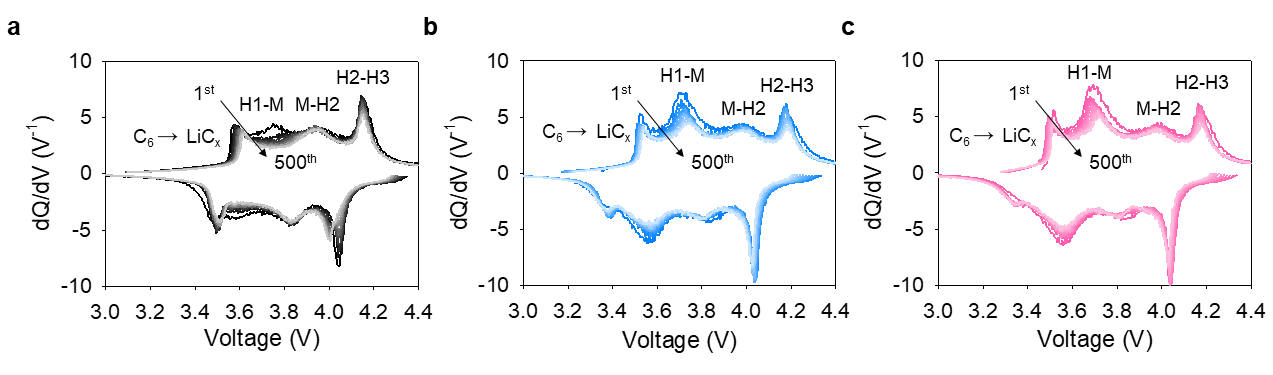


**Figure S52.** Charge/discharge behaviors and corresponding dQ/dV plots for the 1, 50, 100, 250, 300, 350, 400, 450, and 500 cycles of NCM811/graphite full cells: a) baseline, b) 1% VC, and c) 1% ISDMS electrolytes. For the cycle condition, the cycle rate was 1 C/0.5 C, and the temperature was 25 ℃.

**Supporting references**

[1] B. Delley, *J Chem Phys.* **1990**, *92*, 508.

[2] B. Delley, *J. Chem. Phys.* **2000**, *113*, 7756.

[3] P. M. W. Gill, B. G. Johnson, J. A. Pople, M. J. Frisch, Chem. *Phys. Lett.* **1992**, *197*, 499.

[4] H. Looyenga, *Physica*, **1965**, *31*, 401.

[5] R. L. C. Akkermans, N. A. Spenley, S. H. Robertson, *Mol. Simul.* **2021**, *47*, 540.

[6] B. A. Wells, A. L. Chaffee, *J. Chem. Theory Comput.* **2015**, *11*, 3684.

[7] T. H. Wan, M. Saccoccio, C. Chen, F. Ciucci, *Electrochim Acta* **2015**, *184*, 483.

[8] J. P. Schmidt, P. Berg, M. Schönleber, A. Weber, E. Ivers-Tiffée, *J Power Sources* **2013**, *221*, 70.

[9] J. Illig, T. Chrobak, D. Klotz, E. Ivers-Tiffée, *ECS Trans,* **2011**, *33*, 3.

[10] S. Park, S. Y. Jeong, T. K. Lee, M. W. Park, H. Y. Lim, J. Sung, J. Cho, S. K. Kwak, S. Y. Hong, N. –S. Choi, *Nat Commun*. **2021**, *12*, 838.

[11] C. Sun, X. Ji, S. Weng, R. Li, X. Huang, C. Zhu, X. Xiao, T. Deng, L. Fan, X. Wang, C. Wang, X. Fun, *Adv. Mater*. **2022**, *34*, 2206020.

[12] D. J. Kautz, X. Cao, P. Gao, B. E. Matthews, Y. Xu, K. S. Han, F. Omenya, M. H. Engelhard, H. Jia, C. Wang, J.-G. Zhang, W. Xu, *Adv. Energy Mater*. **2023**, *13*, 2301199.

[13] J. G. Han, M. Y. Jeong, K. Kim, C. Park, C. H. Sung, D. W. Bak, K. H. Kim, K. M. Jeong, N. -S. Choi, *J Power Sources* **2020**, *446*, 227366.

[14] X. Xu, X. Yue, Y. Chen, Z. Liang, *Angew. Chem. Int. Ed.* **2023***, 62*, e202306963.

[15] X. Yue, J. Zhang, Y. Dong, Y. Chen, Z. Shi, X. Xu, X. Li, Z. Liang, *Angew. Chem. Int. Ed.* **2023***, 19*, e202302285.
